# Short-term exposure to nitrogen dioxide and emergency department visits for cause-stroke: a time-series study in Shanghai, China, 2013–2022

**DOI:** 10.1265/ehpm.24-00304

**Published:** 2024-12-07

**Authors:** Yonghong Zhou, Yi Jin, Zheng Zhang

**Affiliations:** 1 Affiliated Renhe Hospital of Shanghai University (Renhe Hospital, Baoshan District), School of Medicine, Shanghai University, Shanghai, China; 2 Department of Radiation Oncology, Hunan Cancer Hospital, The Affiliated Cancer Hospital of Xiangya School of Medicine, Central South University, Changsha, Hunan, China; 3 Service of Endocrinology, Affiliated Renhe Hospital of Shanghai University (Renhe Hospital, Baoshan District), Shanghai, China

**Keywords:** Air pollution, Nitrogen dioxide, Stroke, Emergency department visits, Time-series study

## Abstract

**Background:**

The association between air pollution and an increased risk of cardiovascular diseases, including stroke, is well-established. However, it remains unclear how reductions in pollutant levels—resulting from clean air policies and the COVID-19 lockdown—affect this relationship.

**Methods:**

A time-series study was conducted using data from Shanghai, China, spanning from 2013 to 2022, divided into two periods (2013–2019 and 2020–2022). Daily air pollution data were obtained from China’s air quality platform, while stroke emergency department (ED) visits were sourced from Renhe Hospital. We employed quasi-Poisson regression to analyze the relationship between daily pollutant levels and stroke ED visits, with stratified analyses by sex, age, season, and period. The study identified significant reductions in six pollutants (PM_2.5_, PM_10–2.5_, PM_10_, SO_2_, NO_2_, CO) during the 2020–2022 period compared to 2013–2019.

**Results:**

Significant reductions in six air pollutants (NO_2_, PM_2.5_, PM_10–2.5_, PM_10_, SO_2_, CO) were observed during 2020–2022 compared to 2013–2019. Higher daily NO_2_ levels were associated with an increased risk of stroke and its subtypes throughout the study, with a stronger correlation observed in the 2020–2022 period (*P* < 0.001). Subgroup analyses indicated that females and individuals aged 65–74 experienced the highest risks. The elevated stroke risk was particularly pronounced in the summer during 2020–2022. A two-factor model demonstrated that combined exposure to NO_2_ and other pollutants increased stroke risk.

**Conclusions:**

This study heightened that reduced NO_2_ levels generally mitigate the adverse effects of short-term exposure to air pollutants on stroke risk, although the benefits vary among subgroups. The persistent stroke risk despite lower pollutant levels underscores the complex factors influencing stroke risk, highlighting the need for comprehensive intervention strategies.

**Supplementary information:**

The online version contains supplementary material available at https://doi.org/10.1265/ehpm.24-00304.

## 1. Introduction

Stroke represents a predominant cause of disability and mortality on a global scale, imposing considerable burdens on both individuals and society at large. The incidence and mortality rates associated with stroke demonstrate significant geographical variations, with low- and middle-income countries experiencing a disproportionately higher burden. It is estimated that stroke accounts for over 6 million deaths annually worldwide, positioning it among the top ten causes of death globally [1]. Furthermore, more than half of stroke survivors are likely to encounter some form of disability, which carries long-term implications for their daily living and occupational capabilities, thereby exacerbating the strain on healthcare systems [2].

Air pollution, particularly nitrogen dioxide (NO_2_), has been recognized as a significant environmental risk factor for the incidence of stroke. Research indicates that both long-term and short-term exposure to elevated levels of air pollution is closely linked to an increased risk of stroke [3–5]. The proposed underlying mechanisms include systemic inflammation, oxidative stress, and endothelial dysfunction, which are crucial in the pathogenesis of atherosclerosis and thrombosis [6–8], both of which are integral to the development of ischemic stroke. Specifically, NO_2_, a byproduct of fossil fuel combustion and a marker of traffic-related air pollution, has garnered attention due to its ability to penetrate deeply into the lungs, thereby impairing respiratory and cardiovascular functions [9]. Consequently, investigating the effects of NO_2_ on ischemic stroke is essential not only for elucidating the pathophysiological mechanisms involved but also for guiding public health policies and interventions aimed at mitigating stroke risk.

The emergence of the COVID-19 pandemic, accompanied by widespread global lockdowns, provided an unintentional opportunity to investigate the effects of markedly diminished human activities on air quality. Preliminary evidence indicates that these lockdown measures have resulted in significant reductions in the concentrations of several critical air pollutants, including NO_2_ [10, 11]. This situation offers a distinctive opportunity to evaluate the influence of enhanced air quality on public health outcomes, particularly the incidence of stroke, within a relatively brief period.

This study seeks to address a gap in the existing literature by investigating the short-term exposure to NO_2_ and its correlation with emergency department (ED) visits for stroke and its three types during 2013–2022 in Shanghai, China. Our analysis will consider potential differences among various population subgroups, thereby offering insights into the development of future strategies for the control and prevention of stroke.

## 2. Material and methods

### 2.1 Study design and setting

Our study used a time-series study design, which was developed to evaluate the short-term effects of risk factors on adverse health outcomes by utilizing the exposure data of study subjects from different time [12]. The study was conducted in Shanghai, China, a region characterized by a subtropical monsoon climate, featuring hot and rainy summers alongside cold and relatively dry winters [13]. The study period extended from January 1, 2013, to December 31, 2022, facilitating a comparative analysis of stroke and its three types across varying pollutant levels during two distinct periods (2013–2019 *vs.* 2020–2022). We compared pollution levels across the two periods, utilizing data on seven air pollutants (PM_2.5_, PM_10–2.5_, PM_10_, SO_2_, NO_2_, CO, and O_3_-8h) as documented by the local government.

### 2.2 Data on ED visits

Data on ED visits were obtained from Renhe Hospital, located in the Baoshan District of Shanghai, China. This hospital operates a full-service ED staffed with full-time physicians available 24 h/day and serves an overall catchment population of approximately 2 million individuals. The detailed elements of ED visit data recorded included visit dates, age, sex, and discharge diagnoses. All discharge diagnoses were meticulously coded by a seasoned medical record nosologist. The tenth edition of the International Classification of Diseases (ICD-10) was utilized to identify patients with discharge diagnoses of stroke (I60–I90), transient cerebral ischemic attacks, and related syndromes (TIA, G45). Furthermore, stroke cases were categorized into hemorrhagic stroke [(I60–I69), excluding I63] and ischemic stroke (I63) [14]. The process for identifying stroke cases from the ED database is illustrated in a flow diagram (Fig. S1).

### 2.3 Air pollution and meteorological data

Data on seven air pollutants were calculated using hourly concentration data sourced from the air quality sharing platform managed by the Ministry of Environmental Protection [15]. For ozone, the 8-h maximum value (O_3_-8h) was used. This platform provides real-time concentrations of criteria air pollutants from all national air quality monitoring sites across China [16, 17]. We employed the average concentrations of air pollutants from all available monitoring stations in Shanghai as a proxy for assessing air pollution exposure. In addition, daily mean temperature (°C) and relative humidity (%) data were obtained from the National Weather Data Sharing System of China (http://data.cma.cn) [16].

### 2.4 Quantification and statistical analysis

We used the Chi-square test or the Wilcoxon rank-sum test, as appropriate, to compare air pollution and meteorological data. A comprehensive investigation was conducted to explore the associations between stroke-related ED visit frequency and short-term air pollution exposure [16]. Using a GAM and meteorological variables as covariates, the association between daily variations in pollutant levels and stroke-related ED visit was evaluated (Further details refer to the supplemental methods).

The primary model examined moving average exposure spanning from the present day to the preceding 1 to 10 days (lag01-010) and single-day lags from the current day to seven days prior (lag0-7). The maximum estimates and the minimum *P*-values were used to identify the optimal lag periods [18]. The estimates with a corresponding 95% confidence interval (95% CI) were reported as excess risk (ER). These values show the percentage change in morbidity associated with an increase in different air pollutants of 10 units [19]. Subsequently, an exposure-response curve was used to show the correlation between NO_2_ concentrations and the number of stroke-related ED visit. This curve was generated employing a methodology previously studies [16, 20].

To investigate the potential differential effects of NO_2_ exposure on stroke risk among various subgroups, stratified analyses were performed based on sex (male and female), age categories (<55, 55–64, 65–74, 75–84, and ≥85 years), season (warm season: April to September; cold season: October to March), and periods of varying pollutant levels. A Cochrane Q test was employed to assess statistically significant differences in risk estimates across the different strata.

Sensitivity analyses were performed to evaluate the robustness of our findings across various model specifications and choices of smoothing parameters. In these analyses, the degrees of freedom (df) for the calendar time range of 4 to 16, the average temperature range of 4 to 8, and the relative humidity range of 3 to 5 were modified. Furthermore, to investigate the combined effects of NO_2_ and other pollutants on the risk of stroke, we developed a two-factor interaction model. This model facilitates the assessment of both interactive and additive effects of multiple pollutants on the incidence of stroke. In our analysis, we used moving average temperatures for the current day along with the previous three days (temp03), the previous seven days (temp07), and the previous fourteen days (temp014) as proxies for the average temperature of the current day in order to account for the influence of ambient temperature over an extended period of time [16].

R software (version 4.3.2) equipped with the ‘mgcv’ package for GAMs. An association was deemed statistically significant when the two-sided *P*-value was less than 0.05.

## 3. Results

### 3.1 Description of the study population

We enrolled 301,788 stroke-related ED visit in this study (Table 1, Fig. S1). Excluding the hospital lockdown period from April 1, 2022, to June 30, 2022, which was instituted in response to the COVID-19 pandemic, the time series analysis of ED visits indicated a reduction in the variability of stroke-related visits from 2013 to 2022. This period was also characterized by a diminished seasonal trend (Fig. S2). Ischemic strokes represented the most prevalent type of stroke diagnoses, with a total of 151,042 cases, accounting for over half of all stroke-related ED visits. The number of daily ED visits for stroke types varied across different sex, age groups, seasons, and periods (*P* ≤ 0.001). Notably, 92.81% of stroke-related ED visits were from patients aged 55 years or older, with the age group of 65–74 exhibiting the highest percentage of stroke visits at 32.6%. The findings indicate a higher prevalence of stroke among women (55.84%) and during the “cool” seasons (51.31%). Furthermore, the demographic characteristics of ischemic and hemorrhagic strokes were comparable to those of the overall stroke. However, a significant majority of TIA patients were male (64.74%).

**Table 1:** Demographic characteristics of ED visits with stroke in Shanghai city, 2013–2022.

	**Stroke**	**Hemorrhagic stroke**	**Ischemic stroke**	**TIA**	*P* value*
**Sex**					<0.001
Male	133,270 (44.16)	29,851 (46.76)	72,778 (48.18)	30,641 (35.26)	
Female	168,518 (55.84)	33,987 (53.24)	78,264 (51.82)	56,267 (64.74)	
**Age group (years)**					<0.001
<55	21,699 (7.19)	5,974 (9.36)	6,320 (4.18)	9,405 (10.82)	
55–64	62,220 (20.62)	14,366 (22.5)	27,481 (18.19)	20,373 (23.44)	
65–74	98,391 (32.6)	19,605 (30.71)	49,294 (32.64)	29,492 (33.93)	
75–84	76,912 (25.49)	15,054 (23.58)	43,007 (28.47)	18,851 (21.69)	
≥85	42,566 (14.1)	8,839 (13.85)	24,940 (16.51)	8,787 (10.11)	
**Season**					0.001
Warm	146,937 (48.69)	31,129 (48.76)	73,069 (48.38)	42,739 (49.18)	
Cold	154,851 (51.31)	32,709 (51.24)	77,973 (51.62)	44,169 (50.82)	
**Period**					<0.001
1^st^ group (2013–2019)	189,123 (62.67)	45,531 (71.32)	87,450 (57.90)	56,142 (64.60)	
2^nd^ group (2020–2022)	112,665 (37.33)	18,307 (28.68)	63,592 (42.10)	30,766 (35.40)	
**Total**	301,788	63,838	151,042	86,908	

Note: Data are represented as No (%). From March to June 2022, designated COVID-19 treatment hospitals only admitted patients with fever or COVID-19, leading to a sharp decrease in other cases during this period. *A Chi-square test was used to test the difference between Hemorrhagic stroke, Ischemic stroke and TIA.

### 3.2 Air quality and meteorological conditions

Figure S3 illustrates the temporal patterns of various air pollutants throughout the study period, whereas Table 2 presents data on temperature, relative humidity, and the concentrations of the different air pollutants. Overall, distinct seasonal trends are evident for all air contaminants. While several pollutants demonstrated elevated concentrations during the colder months, the concentrations of O_3_-8h were notably higher during the summer season. The “WHO Air quality guidelines” concentration limits were exceeded by the daily average concentrations of NO_2_, PM_2.5_ and PM_10_ [21].

**Table 2:** Summary statistics of daily air pollution and meteorological factors in Shanghai during 2013–2022

	**Mean**	**SD**	**Minimum**	**Q1**	**Median**	**Q3**	**Maximum**
NO_2_ (µg/m^3^), n = 3,619	39.31	19.05	4.09	25.29	35.50	49.50	140.08
PM_2.5_ (µg/m^3^), n = 3,618	40.39	29.83	3.58	19.92	32.11	51.47	252.98
PM_10–2.5_ (µg/m^3^), n = 3,285	19.54	16.15	2.13	10.33	15.95	23.43	193.21
PM_10_ (µg/m^3^), n = 3,618	57.62	36.91	6.96	33.22	47.52	71.01	309.88
O_3_-8h (µg/m^3^), n = 3,600	92.68	42.01	6.18	62.30	87.88	115.88	280.00
SO_2_ (µg/m^3^), n = 3,617	11.62	9.42	3.75	6.00	9.04	13.48	106.90
CO (mg/m^3^), n = 3,583	0.72	0.25	0.31	0.55	0.66	0.82	2.31
Mean temperature (°C), n = 3,622	17.93	8.76	−5.98	10.35	18.65	25.11	35.51
Relative humidity (%), n = 3,622	71.55	12.93	32.69	62.79	71.66	81.09	99.95

Note: SD means standard deviation, Q1 means first quartile, Q3 means third quartile.

**Fig. 1:**
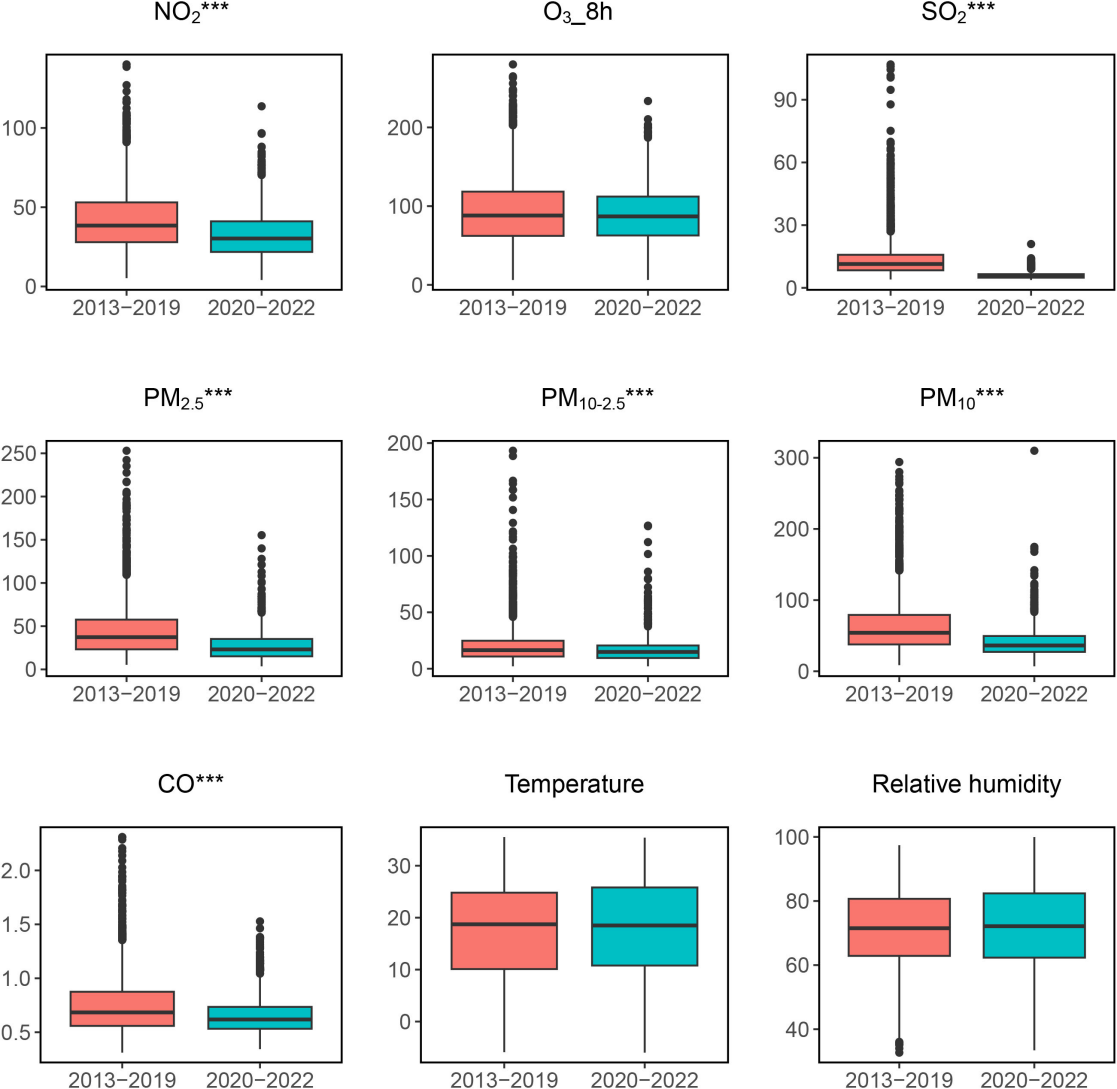
The comparison of air pollutants and meteorological parameters between 2013–2019 and 2020–2022. The thick line in the box represents the median (Q2), the upper bound of the box represents the upper quartile (Q3), and the lower bound of the box represents the lower quartile (Q1). The end of the line above the box represents the upper limit (Q3 + 1.5 × IQR), while the end of the line below the box represents the lower limit (Q1-1.5 × IQR). The points outside the box represent extreme values. ***P < 0.001.

An analysis of air quality data from Shanghai spanning the years 2013 to 2022 reveals significant reductions in several key pollutants during the 2^nd^ period (2020–2022) when compared to 1^st^ period (2013–2019). The improvements in air quality during this timeframe are illustrated in Fig. 1, which clearly demonstrates a decrease in pollutant levels, including NO_2_, PM_2.5_, PM_10–2.5_, PM_10_, SO_2_ and CO, is apparent (*P* < 0.001). These changes are indicative of the effects of COVID-19 lockdown measures and the implementation of clean air policies.

Results of spearman’s correlations presented that NO_2_ exhibited a moderate association with CO (r = 0.727, *P* < 0.001), PM_2.5_ (r = 0.637, *P* < 0.001), PM_10_ (r = 0.611, *P* = 0.005), SO_2_ (r = 0.453, *P* = 0.007) and mean temperature (r = −0.401, *P* < 0.001). Meanwhile, the associations between NO_2_, PM_10–2.5_, O_3_-8h, and relative humidity were found to be insignificant (Fig. S4 and Table S1).

### 3.3 Single pollutant model

**Fig. 2:**
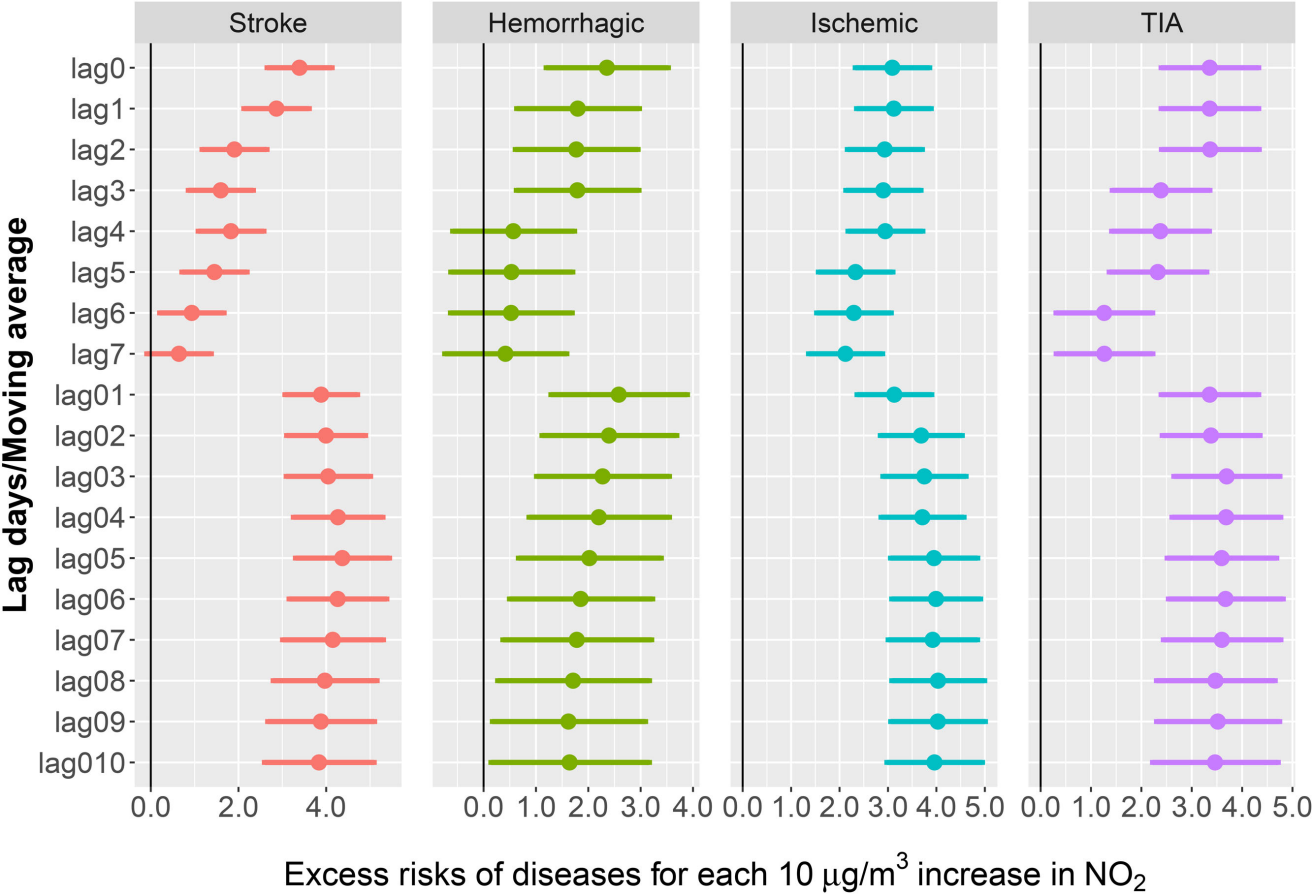
Stroke-related ED visit per 10 µg/m^3^ increase in NO_2_ concentrations. The ER and corresponding 95% CI were derived from an over-dispersed generalized additive model, with calendar time, weather conditions, day of the week (DOW) and public holiday (PH) controlled.

Overall, our data indicate that, particularly in hemorrhagic patients, a 10-unit rise in several ambient air pollutants, including PM_2.5_, PM_10_, PM_10–2.5_, SO_2_, and O_3_-8h, is associated with a lower risk of stroke-related ED visit. In contrast, an increase in NO_2_ levels is observed (Fig. 2 and Fig. S5). At lag0-6, and lag01-010, NO_2_ exhibited a significant correlation with the frequency of stroke-related ED visit, with the strongest estimates at lag05 (ER: 4.37%, 95% CI: 3.30%, 5.44%). Similarly, robust positive correlations were identified between NO_2_ and the incidence of ischemic strokes, hemorrhagic strokes, and TIA. It is noteworthy that the effect ratio for stroke per 10 µg/m^3^ increase in NO_2_ concentration was markedly elevated during the pandemic years, suggesting an increased vulnerability to NO_2_ exposure during this period (Fig. S6).

**Fig. 3:**
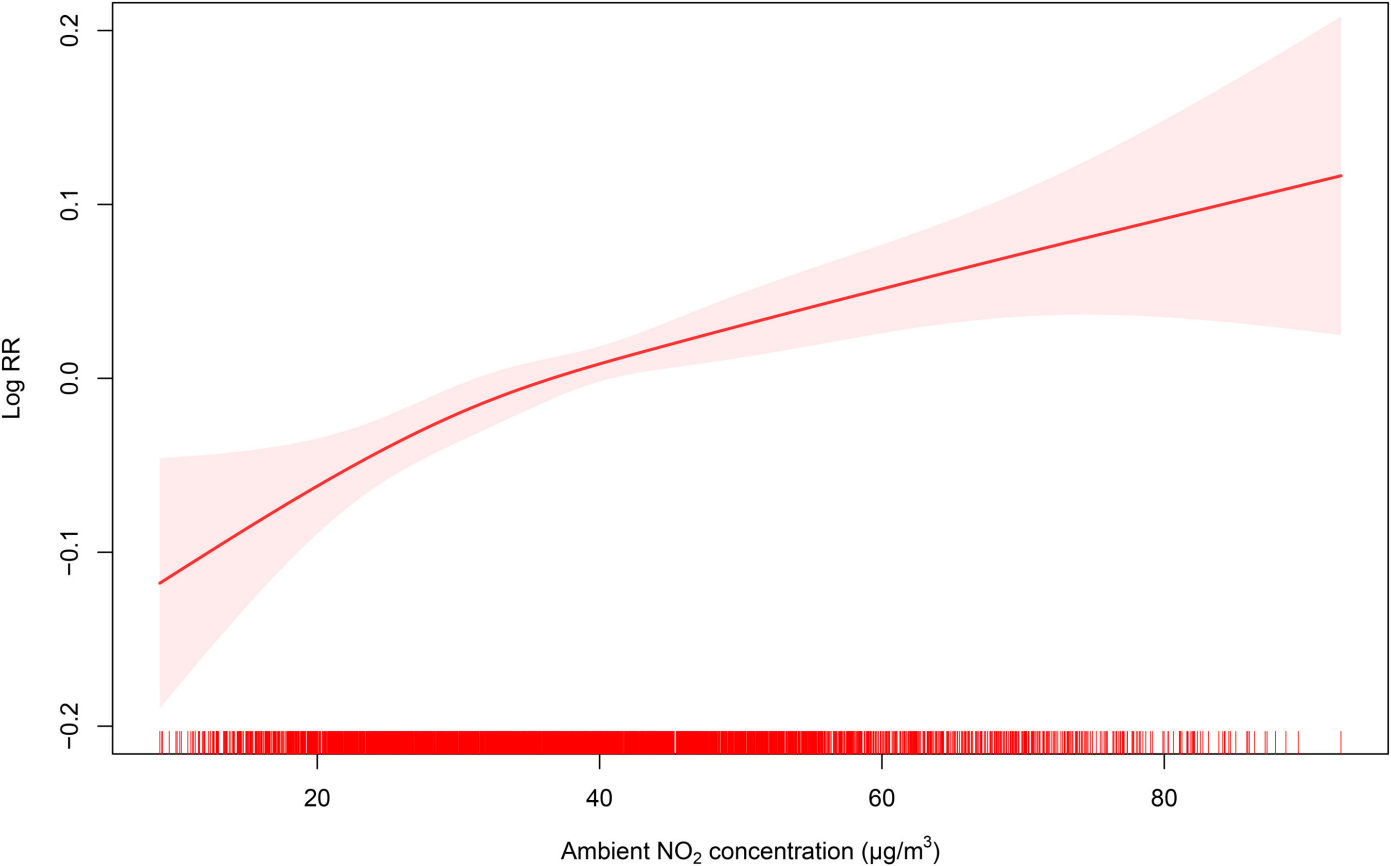
Exposure-response curve for the association between NO_2_ (lag05) and stroke-related ED visit. The line represents the point estimates and the shading indicates corresponding 95% CIs, which were derived from an over-dispersed generalized additive model, with calendar time, weather conditions, day of the week (DOW) and public holiday (PH) controlled.

As displayed in Fig. 3 and S7 illustrate the virtually linear decline in the number of stroke-related ED visit. Regarding O_3_-8h, we found that there was a little initial rise in the number of ED visits linked to stroke, which peaked at specific air pollution levels of 55.96 µg/m^3^, followed by a subsequent decrease. However, the data indicate that the number of stroke-related ED visits exhibited appropriately linear correlations with various air pollutants, including NO_2_, PM_2.5_, PM_10-2.5_, PM_10_, and CO, suggesting that higher concentrations of these pollutants were associated with an increase in ED visits. Notably, as the concentrations of these contaminants increased, no discernible thresholds were identified. Furthermore, differences were observed in the exposure-response curve concerning the relationship between NO_2_ levels and the number of stroke-related ED visits across the two periods, as depicted in Fig. S8. Specifically, we noted that the number of stroke-related ED visits decreased to a minimum value at 42.22 µg/m^3^ of NO_2_, after which it increased, exhibiting a flat slope at both low and high concentration ranges. Due to the small difference of the ER for NO_2_ between the larges estimates value and the value at lag0 for stroke and its subtypes, we concentrated on the effects of NO_2_ (lag0) for our subsequent analyses for unity.

### 3.4 Stratified analysis

Table 3 summarizes the findings of the stratified analyses. When the data were divided by sex, we observed that the associations between NO_2_ and stroke were weaker in men than in women. A similar trend was observed in TIA, but the opposite tendency was seen in hemorrhagic and ischemic strokes.

**Table 3:** Summary statistics of the stratified analyses

	**Stroke**	**Hemorrhagic stroke**	**Ischemic stroke**	**TIA**
**Gender**				
Male	3.07 (2.29, 3.85)***	2.10 (0.90, 3.32)***	2.93 (2.11, 3.75)***	2.11 (1.05, 3.18)***
Female	3.51 (2.75, 4.27)***	2.07 (0.83, 3.33)**	2.87 (2.06, 3.68)***	3.48 (2.48, 4.50)***
**Age group (years)**				
<55	1.40 (0.29, 2.52)*	1.03 (−0.45, 2.53)	0.13 (−1.31, 1.59)	0.53 (−0.93, 2.01)
55–64	2.52 (1.70, 3.36)***	1.92 (0.64, 3.22)**	1.97 (1.00, 2.94)***	1.71 (0.55, 2.89)**
65–74	3.47 (2.65, 4.29)***	2.32 (1.08, 3.57)***	2.57 (1.68, 3.47)***	3.40 (2.34, 4.48)***
75–84	3.02 (2.20, 3.85)***	1.92 (0.45, 3.41)*	2.52 (1.64, 3.40)***	1.96 (0.79, 3.15)**
≥85	2.84 (1.93, 3.76)***	1.45 (−0.00, 2.92)	2.31 (1.32, 3.31)***	1.97 (0.61, 3.36)**
**Season**				
Warm	2.99 (1.46, 4.55)***	−0.22 (−2.41, 2.02)	2.96 (1.33, 4.61)***	3.06 (1.22, 4.93)**
Cold	2.62 (1.80, 3.44)***	2.49 (1.13, 3.86)***	2.23 (1.40, 3.07)***	2.93 (1.73, 4.13)***
**Period**				
1^st^ group (2013–2019)	2.14 (1.36, 2.92)***	1.86 (0.47, 3.27)**	1.71 (0.87, 2.55)***	2.61 (1.46, 3.77)***
2^nd^ group (2020–2022)	5.44 (3.77, 7.15)***	5.64 (3.64, 7.67)***	5.10 (3.39, 6.84)***	4.88 (3.08, 6.72)***
**Total**	3.39 (2.65, 4.13)***	2.36 (1.20, 3.53)***	3.09 (2.33, 3.86)***	3.36 (2.39, 4.33)***

Note: ER(%) and 95% CI of stroke-related ED visit associated with a 10 µg/m^3^ increase in NO_2_ (lag0) concentrations stratified by sex, age and season. Associations of statistically significance are listed in the table, ***, ** and * indicate p < 0.001, p < 0.01 and p < 0.05, respectively. For details see the Supplementary Table S2–4.

In the age-specific analyses, NO_2_ was found to have significant associations with stroke and its types-related ED visit across all age groups, with the largest effect observed at 65–74 years. In the season-stratified analysis, it was determined that the effect estimates for stroke, ischemic stroke, and TIA were significantly more pronounced during the cold season compared to the warm season. In contrast, the effect estimates for hemorrhagic stroke were greater during the cold season, we were unable to identify significant effects during the warm season. Furthermore, in the analysis of each study period, the association between NO_2_ and stroke, as well as its subtypes, was significantly more pronounced in the second study period than in the first study period (*P* < 0.001).

### 3.5 Sensitivity analyses

In general, the findings highlight the increased risk of stroke associated with the combination of NO_2_ exposure and other pollutants, particularly PM_2.5_, where warm months show an 8.27% increase in stroke risk, demonstrating the synergistic influence of these pollutants on stroke incidence (Table 4). However, when PM_10–2.5_, O_3_-8h and CO were included for hemorrhagic stroke during the warm season, the results lost their significance.

**Table 4:** Summary statistics of two pollutant models analyses

	**Adjusted for PM_2.5_**	**Adjusted for PM_10–2.5_**	**Adjusted for PM_10_**	**Adjusted for SO_2_**	**Adjusted for O_3_-8h**	**Adjusted for CO^#^**
**Warm**						
Stroke	8.27 (6.16, 10.43)***	2.83 (1.21, 4.48)***	6.24 (4.26, 8.27)***	7.90 (6.07, 9.77)***	5.05 (3.41, 6.72)***	8.73 (6.46, 11.04)***
Hemorrhagic stroke	6.67 (3.61, 9.83)***	−0.36 (−2.61, 1.94)	5.64 (2.74, 8.61)***	2.99 (0.34, 5.71)*	2.32 (−0.03, 4.73)	1.13 (−2.02, 4.37)
Ischemic stroke	7.08 (4.85, 9.36)***	2.64 (0.90, 4.40)**	5.23 (3.14, 7.36)***	8.28 (6.33, 10.28)***	5.06 (3.31, 6.84)***	10.00 (7.58, 12.48)***
TIA	6.83 (4.30, 9.42)***	3.13 (1.16, 5.13)**	5.35 (2.99, 7.77)***	7.75 (5.55, 10.01)***	4.02 (2.04, 6.03)***	5.82 (3.10, 8.61)***
**Cold**						
Stroke	4.18 (3.11, 5.25)***	2.46 (1.60, 3.32)***	3.93 (2.90, 4.98)***	3.62 (2.69, 4.55)***	2.27 (1.44, 3.11)***	4.51 (3.26, 5.78)***
Hemorrhagic stroke	4.54 (2.73, 6.39)***	2.10 (0.63, 3.60)**	3.98 (2.23, 5.77)***	5.68 (4.05, 7.33)***	1.38 (0.06, 2.72)*	4.42 (2.34, 6.54)***
Ischemic stroke	3.90 (2.81, 4.99)***	2.14 (1.25, 3.04)***	3.88 (2.83, 4.95)***	2.85 (1.92, 3.79)***	2.21 (1.36, 3.08)***	3.93 (2.65, 5.21)***
TIA	3.80 (2.23, 5.39)***	2.72 (1.47, 3.98)***	3.36 (1.86, 4.89)***	3.50 (2.13, 4.89)***	2.49 (1.26, 3.72)***	5.27 (3.43, 7.15)***

Note: Excess risk (%) and 95% CI of ED visits with stroke associated with a 10 µg/m^3^ increase in NO_2_ (lag0) concentrations from two pollutant models, by stroke subtype, Shanghai, 2013–2022. “^#^” indicates that excess risk (%) and 95% CI of ED visits with stroke associated with a 10 mg/m3 increase in CO concentrations. Associations of statistically significance are listed in the table, ***, ** and * indicate p < 0.001, p < 0.01 and p < 0.05, respectively.

The estimates exhibited minor variations as the temperature increased from 4 to 8 degrees and the relative humidity rose from 3 to 5 percent, in conjunction with an increase in the duration of calendar time from 4 to 16 days (Table S2–4). The results remained consistent when the ambient temperature was maintained at a regulated level for an extended period (Table S5).

## 4. Discussion

In this study, we main explored the impact of NO_2_ levels on the ED visits of stroke and its subtypes within Shanghai, China, over nearly a decade from 2013 to 2022. This period witnessed a notable reduction in pollutant levels, likely a result of both local clean air initiatives and the widespread lockdowns enforced during the COVID-19 pandemic. Our principal findings were as follows:1) Elevated NO_2_ levels were significantly associated with an increase in stroke-related ED visit and its subtypes. 2) Six air pollutants (NO_2_, PM_2.5_, PM_10_, PM_10–2.5_, SO_2_, and CO) exhibited a continuous downward trend for the whole duration. 3) The reduction in NO_2_ levels did not mitigate their adverse association with the risk of stroke and its subtypes. 4) The impact of lower NO_2_ levels on the risk of stroke and its subtypes in subpopulations was heterogeneous, indicating differential susceptibilities across demographic groups.

Our findings align with increasing evidence suggesting that air pollution is an emerging risk factor for stroke, contributing to approximately 14% of stroke-related deaths globally [22]. The distinction between ischemic and hemorrhagic strokes, as well as their causes, enhances our understanding of who is most at risk and clarifies the specific impact of air pollution on these conditions [22]. Our study contributes to the existing body of knowledge by providing a comprehensive review during a period of significant global change, particularly the COVID-19 pandemic. This pandemic, coupled with the Chinese government’s Three-year Action Plan to Win the Blue Sky Defense War [23], and the impact of reduced production activities and widespread mask-wearing during the epidemic, has resulted in an unprecedented decrease in air pollution levels. This unique situation has presented an opportunity to investigate whether cleaner air could lead to a reduction in pollution-related diseases [22].

Highlighting the pivotal role of air quality in public health, our study reveals that enhanced NO_2_ exposure is correlated with an augmented risk of stroke. The significant reduction in air pollutants, including NO_2_, during the pandemic period aligns with observations by previous studies [11, 24], who reported decreased pollution levels worldwide due to clean air initiatives and lockdown measures. However, our study extends this observation by directly linking these reductions to changes in stroke incidence, a connection not thoroughly explored in their analysis.

Our research corroborates findings from previous studies, hinting the significant health risks posed by NO_2_. Consistent with Shah et al. [3], our results highlight the need for continued efforts to improve air quality and public health policies aimed at reducing air pollutant levels. This is particularly crucial in low-income and middle-income countries, where the burden of stroke and exposure to high levels of pollution are most pronounced [22]. Furthermore, our analysis light on the persistent risk of stroke associated with NO_2_ exposure, even amidst overall lower pollution levels. This underscores the importance of air quality management as a critical component of public health strategies.

Consistent with prior investigations [25, 26], we discerned demographic disparities in pollution susceptibility, accentuating elevated risks among women, older adults, and during colder seasons. Previous studies indicated that age is a crucial risk factor for stroke. Similar with our study, the incidence of stroke is notably higher among the elderly population (particularly those over 65 years old) compared to the younger cohort [27]. As individuals age, the health conditions of their blood vessels and heart typically worsen, thereby exacerbating the risk of stroke. For instance, a reveal that the influence of NO_2_ exposure on stroke is particularly prominent in the elderly population, with a risk increment rate of 0.75% (95% CI: 0.11–1.40%) [27]. Among different sex, the risk of stroke for females significantly increases in old age. This might be associated with hormonal alterations in postmenopausal women, which can impact vascular health and blood pressure, thereby enhancing the risk of stroke. Research indicates that there is a correlation between short-term NO_2_ exposure and the hospitalization rate of female stroke, especially among elderly women [28, 29]. Compounded by the higher prevalence of stroke among the elderly and the longer lifespans of females, who constitute a significant portion of the aging demographic [30]. The interaction between NO_2_ and age is also regarded as a significant aspect of stroke risk. The elderly population exhibits higher vulnerability when exposed to environmental pollution, possibly due to their declining physiological functions and underlying diseases. Short-term exposure to high concentrations of NO_2_ can exacerbate these issues, resulting in higher incidences and hospitalization rates of stroke [31]. Studies have also showed that the incidence of stroke is higher among the elderly population during cold seasons. These phenomenon may be attributed to the interaction between cold temperatures and physiological responses, such as heightened blood pressure and increased cardiac oxygen demand [32]. In cold weather, the concentration of NO_2_ may rise, and this cumulative effect of NO_2_ exposure further elevates the risk of stroke [33], especially among elderly women [34]. Furthermore, the risk of stroke is not only associated with short-term exposure to NO_2_ but also long-term exposure, which may cause cumulative effects. Numerous studies have shown that the incidence of stroke is higher among people who have long lived in highly polluted areas. This emphasizes the significance of NO_2_ exposure in stroke risk across different age groups [33, 35]. In the strategies for stroke prevention and control, the combined effects of age, gender, and environmental factors need to be taken into consideration. Targeted public health intervention measures for high-risk groups, especially elderly women, will contribute to reducing the incidence of stroke.

Our examination of the synergistic effects of NO_2_ and other pollutants on stroke risk provides a direct contrast to single-pollutant models often employed in prior research [4, 36]. The compounded risk, particularly when NO_2_ levels were elevated alongside PM_2.5_ during warmer months with an 8.27% increase in stroke risk, similar trend has been observed among hemorrhagic stroke, ischemic stroke, and TIA, indicates the complex interactions between different air pollutants and their cumulative health impact. NO_2_ is predominantly originated from vehicle exhaust emission and industrial production. PM_2.5_ sources encompass industrial emissions, fuel combustion, vehicle discharges, and natural dust. This study demonstrates that there exists a notable correlation between NO_2_ and PM_2.5_, indicating that the two frequently coexist in the environment and mutually influence one another. In certain circumstances, increasing in NO_2_ might be associated with elevated PM_2.5_ levels, reflecting their common pollution sources, particularly in urban areas. The interaction of NO_2_ and PM_2.5_ may impact stroke could be interpreted as both NO_2_ and PM_2.5_ lead to oxidative stress, generating free radicals and reactive oxygen species. These active substances can inflict damage on cells and tissues, resulting in heightened inflammation. Studies have indicated that NO_2_ can intensify the oxidative stress response caused by PM_2.5_, thereby causing more substantial damage to the vascular endothelium [37]. Secondly, the combined action of NO_2_ and PM_2.5_ can lead to an increase in the release of inflammatory factors within the body. Exposure to NO_2_ and PM_2.5_ will facilitate the aggregation and activation of white blood cells, releasing various inflammatory mediators. This inflammatory response not only affects the function of blood vessels but also may promote the formation of atherosclerosis, thereby escalating the risk of stroke [37, 38]. The synergy of NO_2_ and PM_2.5_ may result in impaired endothelial cell function, which in turn influences the vasomotor function. Injury to endothelial cells will lead to a decreased vasoconstriction ability and an increased risk of thrombosis, which plays a crucial role in the pathogenesis of stroke [37]. In addition, these pollutants can permeate the blood-brain barrier and cause neuroinflammation and nerve cell damage, which may eventually trigger stroke [37, 39]. Multiple epidemiological studies also support an association between the synergy of NO_2_ and PM_2.5_ and the risk of stroke. For example, studies have shown that the impact of PM_2.5_ is more pronounced in high NO_2_ environments, especially in ischemic stroke mortality [38]. Prolonged exposure to these two pollutants may lead to a higher rate of cardiovascular events, including stroke [37]. Furthermore, the interaction between NO_2_ and PM_2.5_ implies that the two may interact through different mechanisms of health effects, suggesting that taking effective intervention measures, the risk of cardiovascular events such as stroke can be effectively reduced.

While our study’s focus on Shanghai may circumscribe the generalizability of our findings. Moreover, the intrinsic ecological bias of time-series studies and reliance on average air pollution data from fixed monitors, rather than individual exposure metrics, may introduce exposure misclassification. Nonetheless, research by Qiu et al. [40] suggests such misclassification minimally impacts results. In addition, due to the limitations of the data sources used in this study, potential confounding factors (e.g., socioeconomic status, access to healthcare) were not included in the analysis model, which may restrict the general applicability of the research findings. Future studies should consider these factors to provide more accurate analysis and interpretation.

## 5. Conclusions

Our study adds the evidence on the health impacts of air pollution, highlighting the persistent risk of stroke associated with NO_2_ exposure even during periods of overall pollution reduction. Our findings emphasize the need for continued vigilance and proactive measures to mitigate air pollution’s health impacts, particularly in vulnerable populations.

## Abbreviations

ED: emergency department
TIA: transient cerebral ischemic attacks and related syndromes
PM_2.5_: particulate matter with an aerodynamic diameter smaller than or equal to 2.5 µm
PM_10–2.5_: coarse particulate matter with aerodynamic diameter between 2.5 and 10 µm
PM_10_: particulate matter with an aerodynamic diameter smaller than or equal to 10 µm
SO_2_: sulfur dioxide
NO_2_: nitrogen dioxide
O_3_-8h: the 8-h maximum value of ozone
CO: carbon monoxide
ER: excess risk
CI: confidence interval
SD: standard deviation

## Supplementary information

**Additional file 1:** Supplementary methods. **Figure. S1.** Case Diagnosis and Inclusion Process Flowchart. **Figure. S2.** Time-series plot of the daily ED visit numbers for stroke during January 1, 2013 to December 31, 2022. **Figure S3.** Time-series plot of air pollution concentrations during January 1, 2013 to December 31, 2022. **Figure S4.** Spearman correlation between air pollutants and weather conditions in Shanghai city during the study period. **Figure S5** Forest plots of association between the increase of different pollutants and stroke risk for cases in 2023–2022. **Figure S6** Forest plots of association between the increase of NO_2_ and stroke risk for cases in 2013–2019 (A) and 2020–2022 (B). We calculated the odds ratios when the daily values of NO_2_ increased by 10 µg/m^3^. **Figure S7.** Exposure-response curve for the association between different pollutants (lag0) and ED visits for stroke in 2013–2022. **Figure S8.** Exposure-response curve for the association between NO_2_ (lag05) and ED visits for stroke in 2013–2019 (A) and 2020–2022 (B). **Table S1.** Spearman correlation between air pollutants and weather conditions in Shanghai city, 2013–2020. **Table S2** Excess risk (%) and 95%CI of outpatients for stroke associated with a 10 µg/m^3^ increase in NO_2_ (lag0) under varying degrees of freedom for the smooth functions of calendar time in single-pollutant models. **Table S3** Excess risk (%) and 95%CI of outpatients for stroke associated with a 10 µg/m^3^ increase in NO_2_ (lag0) under varying degrees of freedom for the smooth functions of temperature in single-pollutant models. **Table S4** Excess risk (%) and 95%CI of outpatients for stroke associated with a 10 µg/m^3^ increase in NO_2_ (lag0) under varying degrees of freedom for the smooth functions of relative humidity in single-pollutant models. **Table S5** Excess risk (%) and 95% CI of outpatients for stroke associated with a 10 µg/m^3^ increase in NO_2_ (lag0) when a longer time period of ambient temperature was controlled.

## Declarations

### Availability of data and material

All data generated or analysed during this study are included in this published article and its supplementary information files. All other data can be obtained from the corresponding author upon request.
