# Supplementary material for: Short-term exposure to nitrogen dioxide and emergency department visits for cause-stroke: a time-series study in Shanghai, China, 2013–2022
Source: Environ Health Prev Med. 2024 Dec 7;29:67. doi: 10.1265/ehpm.24-00304 (PMC11631555; doi:10.1265/ehpm.24-00304)
Supplement: Supplementary file 1 — Additional file 1: Supplementary methods. Figure. S1. Case Diagnosis and Inclusion Process Flowchart. Figure. S2. Time-series plot of the daily ED visit numbers for stroke during January 1, 2013 to December 31, 2022. Figure S3. Time-series plot of air pollution concentrations during January 1, 2013 to December 31, 2022. Figure S4. Spearman correlation between air pollutants and weather conditions in Shanghai city during the study period. Figure S5 Forest plots of association between the increase of different pollutants and stroke risk for cases in 2023–2022. Figure S6 Forest plots of association between the increase of NO2 and stroke risk for cases in 2013–2019 (A) and 2020–2022 (B). We calculated the odds ratios when the daily values of NO2 increased by 10 µg/m3. Figure S7. Exposure-response curve for the association between different pollutants (lag0) and ED visits for stroke in 2013–2022. Figure S8. Exposure-response curve for the association between NO2 (lag05) and ED visits for stroke in 2013–2019 (A) and 2020–2022 (B). Table S1. Spearman correlation between air pollutants and weather conditions in Shanghai city, 2013–2020. Table S2 Excess risk (%) and 95%CI of outpatients for stroke associated with a 10 µg/m3 increase in NO2 (lag0) under varying degrees of freedom for the smooth functions of calendar time in single-pollutant models. Table S3 Excess risk (%) and 95%CI of outpatients for stroke associated with a 10 µg/m3 increase in NO2 (lag0) under varying degrees of freedom for the smooth functions of temperature in single-pollutant models. Table S4 Excess risk (%) and 95%CI of outpatients for stroke associated with a 10 µg/m3 increase in NO2 (lag0) under varying degrees of freedom for the smooth functions of relative humidity in single-pollutant models. Table S5 Excess risk (%) and 95% CI of outpatients for stroke associated with a 10 µg/m3 increase in NO2 (lag0) when a longer time period of ambient temperature was controlled. [file ehpm-29-067-s001.docx]

**Supplementary methods**

**Quantification and statistical analysis**

The relationship between daily different pollutant levels and ED visits for stroke was assessed using a GAM, considering that the daily ED visits exhibited a quasi-Poisson distribution. The model included the average temperature for the current day (the degree of freedom, df = 6), relative humidity for the current day (df = 3), calendar time (df = 8/year), and adjustment for day of the week (DOW) and public holiday (PH) (Guo et al. 2021; Song et al. 2019). The main model of our study is described as follow:

log (*E*(*Yt* )) = *β* * *Zt* + *ns* (*time*, *df* = 8 / *year*) + *ns* (*temperature*, *df* = 6)+ *ns* (*relative humidity*, *df* = 3) + *factor* (*DOW*) + *factor* (*PH*) + *α*

where *E(Yt)* represents the expected ED visits for stroke on day t, *Zt* refers to NO_2_ concentration on day t, time refers to calendar time which was used to control unmeasured long-term trend, temperature and relative humidity refers to average temperature and relative humidity of the current day, respectively, DOW and PH are dummy variables, *β* is the coefficient for *Zt,* ns refers to a natural cubic smooth function, df refers to the degree of freedom, and α is the intercept. We can calculate the Relative Risk by taking logarithm of *β*.


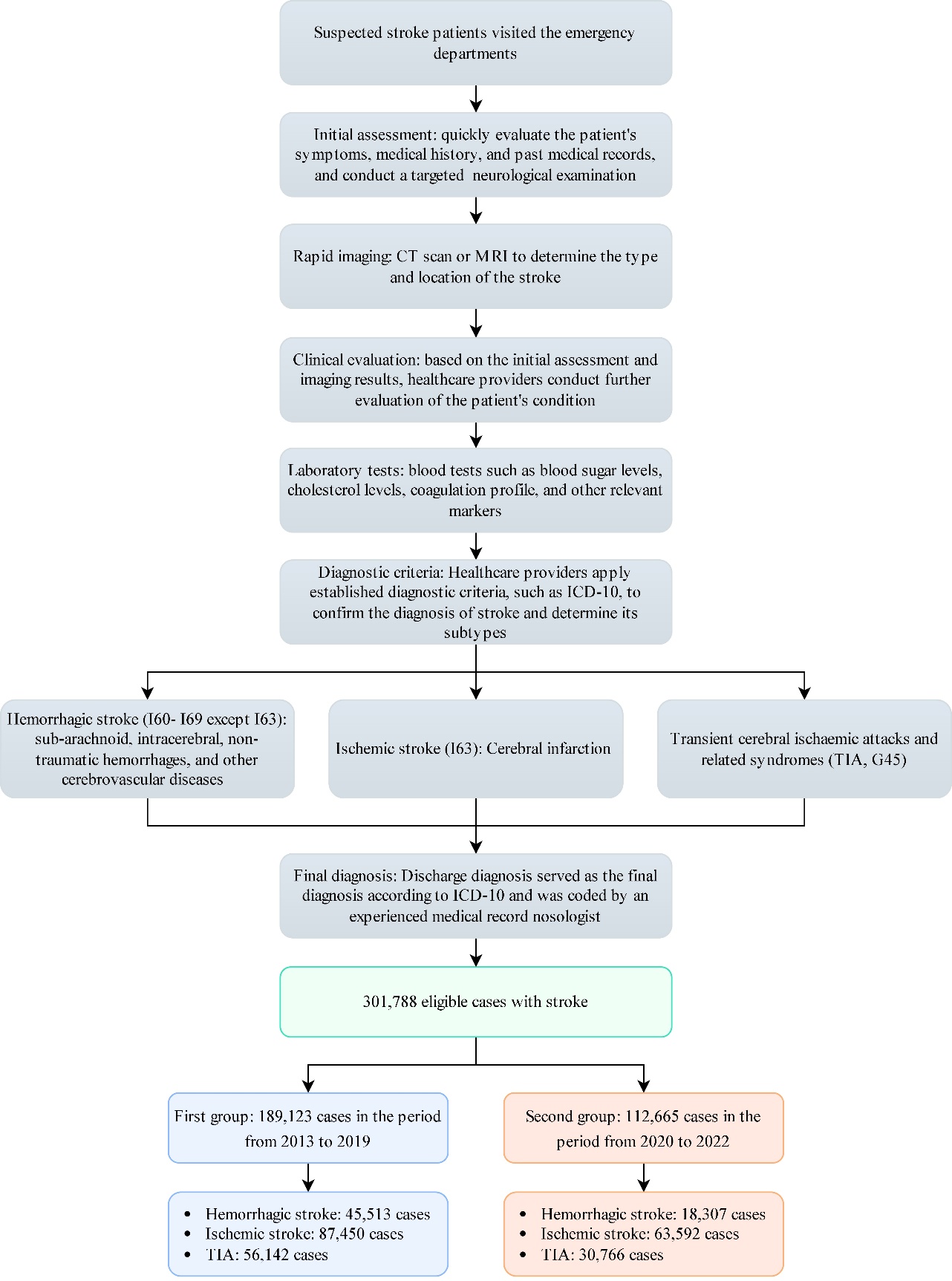


**Figure. S1.** **Case Diagnosis and Inclusion Process Flowchart.**


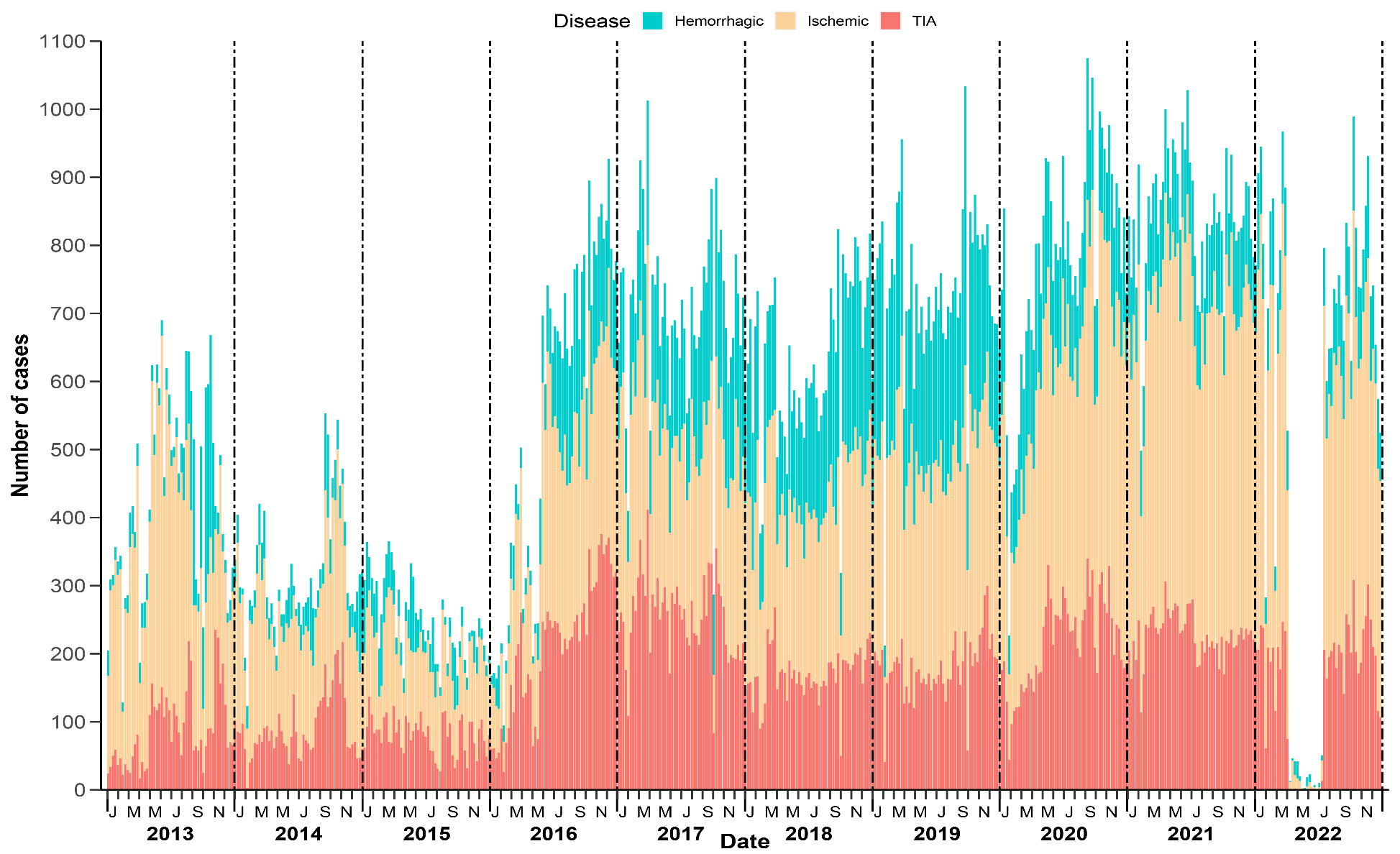


**Figure. S2.** **Time-series plot of the daily ED visit numbers for stroke during January 1, 2013 to December 31, 2022.**


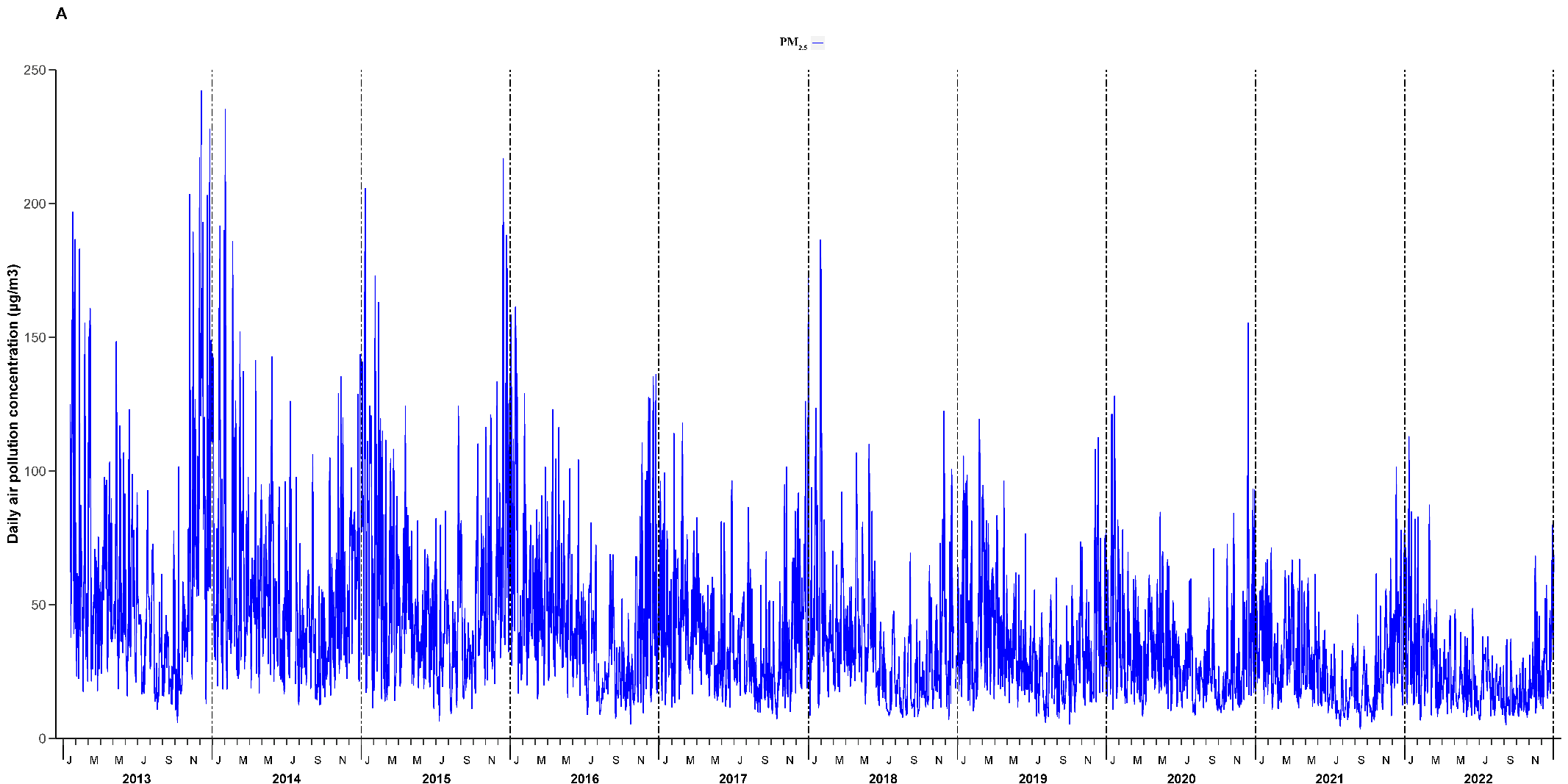

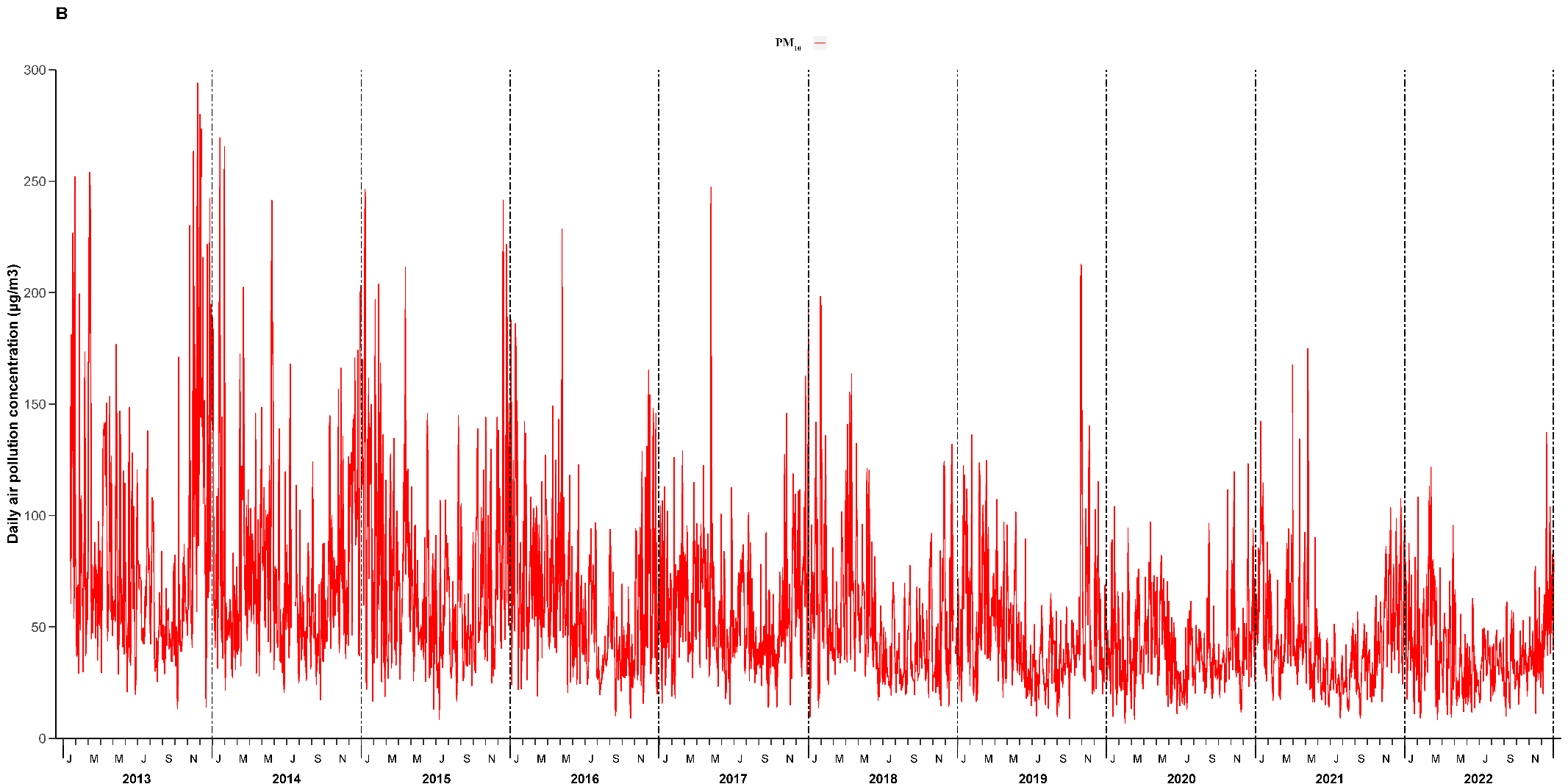

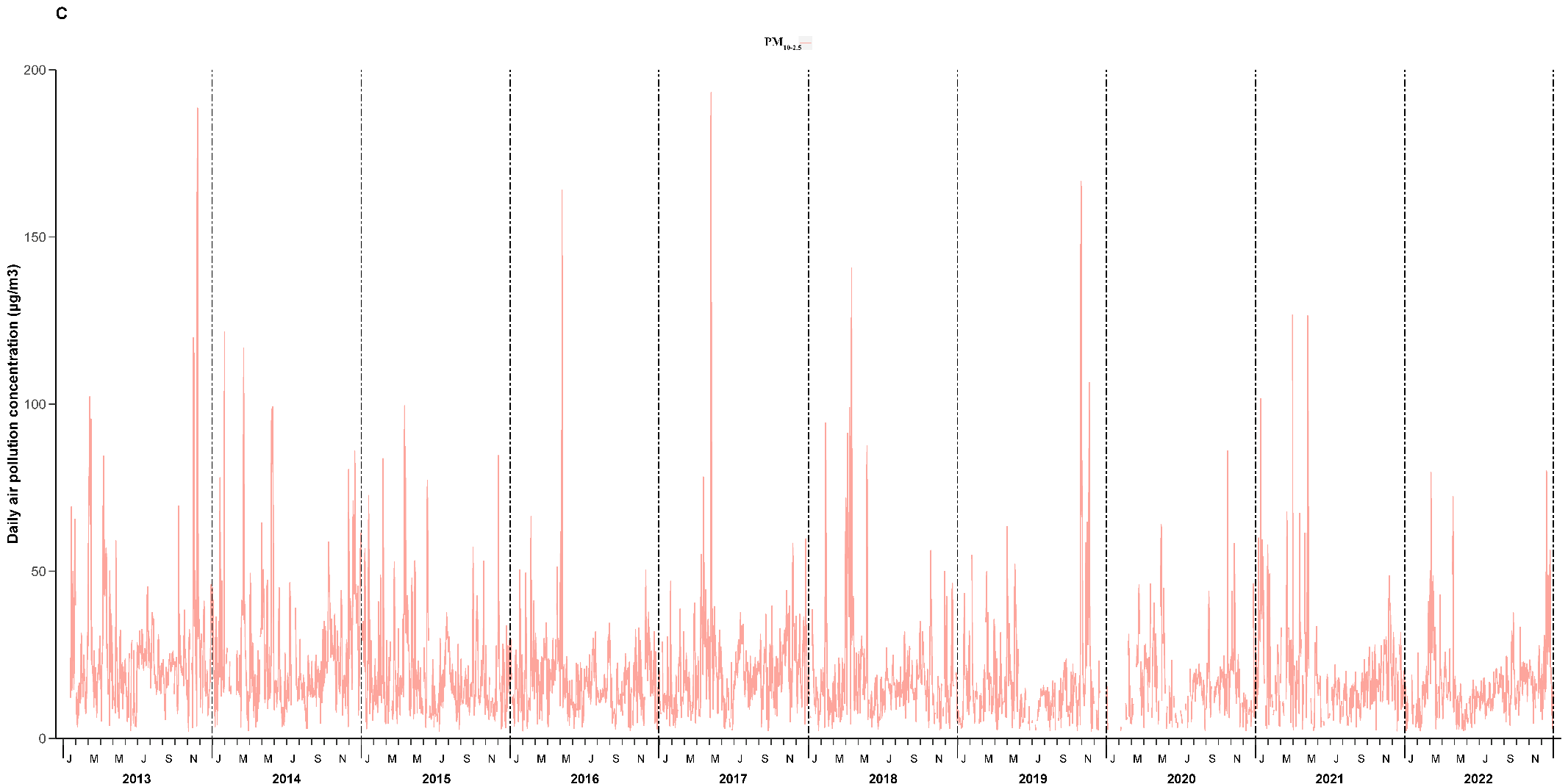

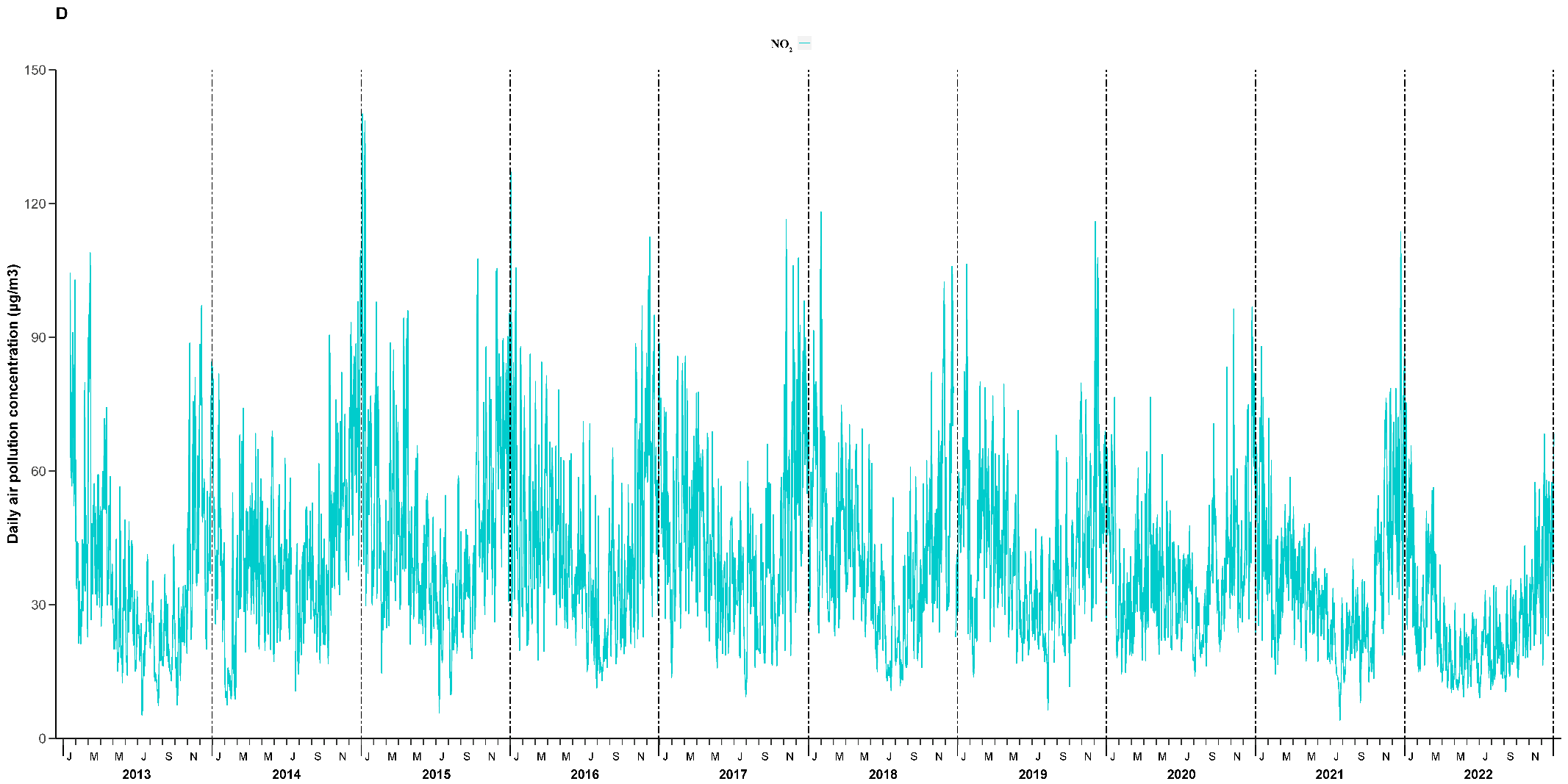

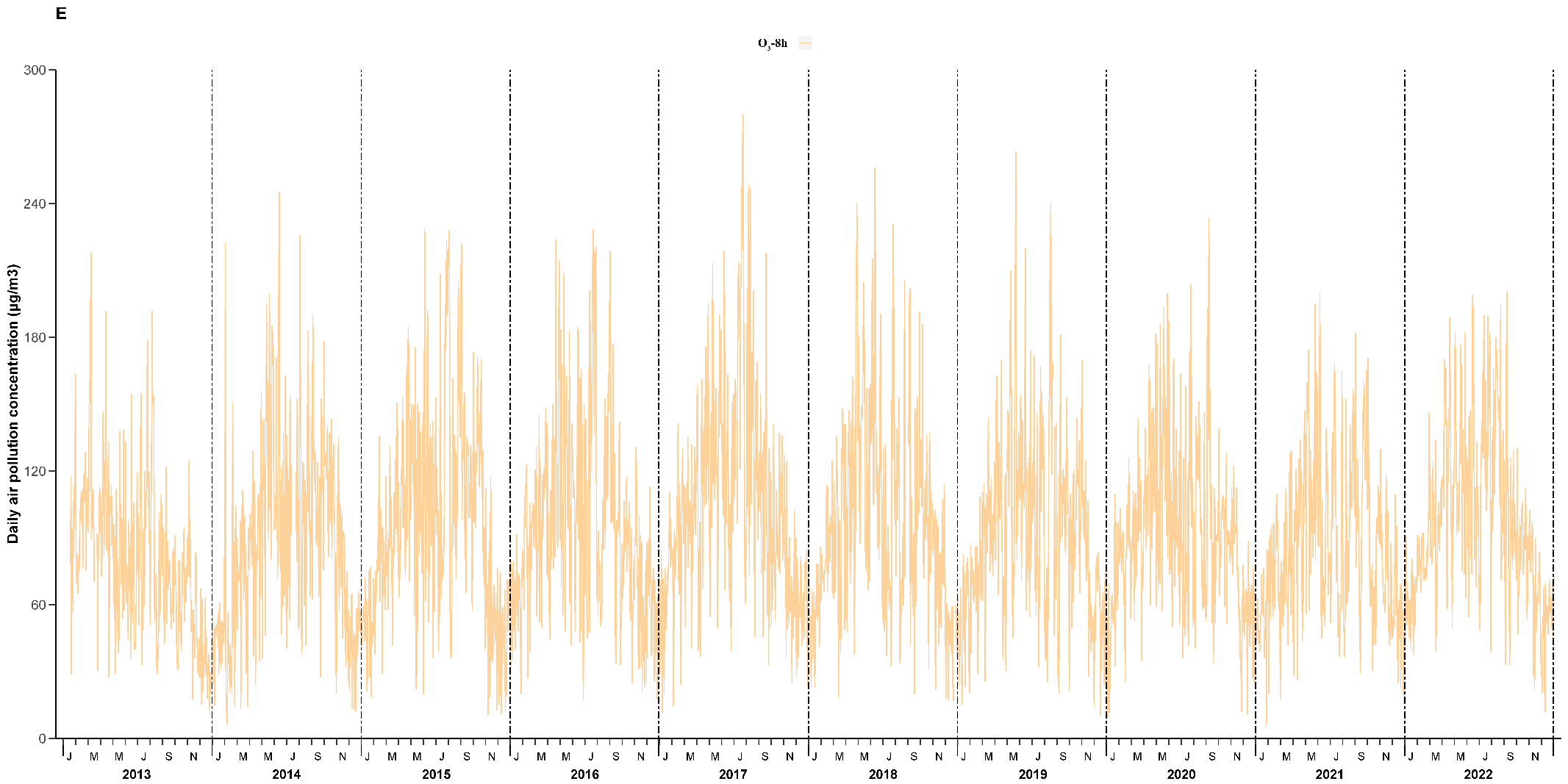

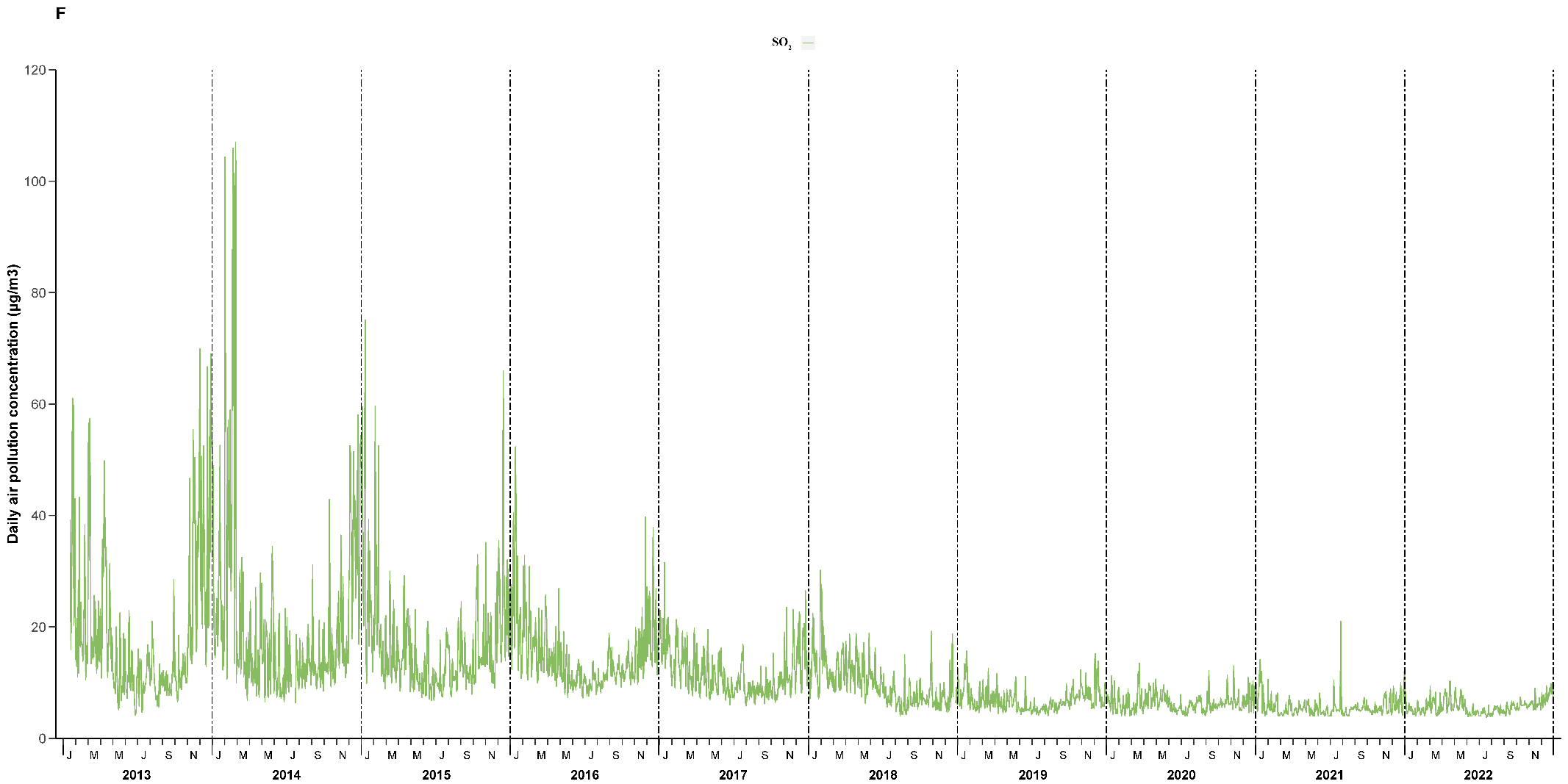

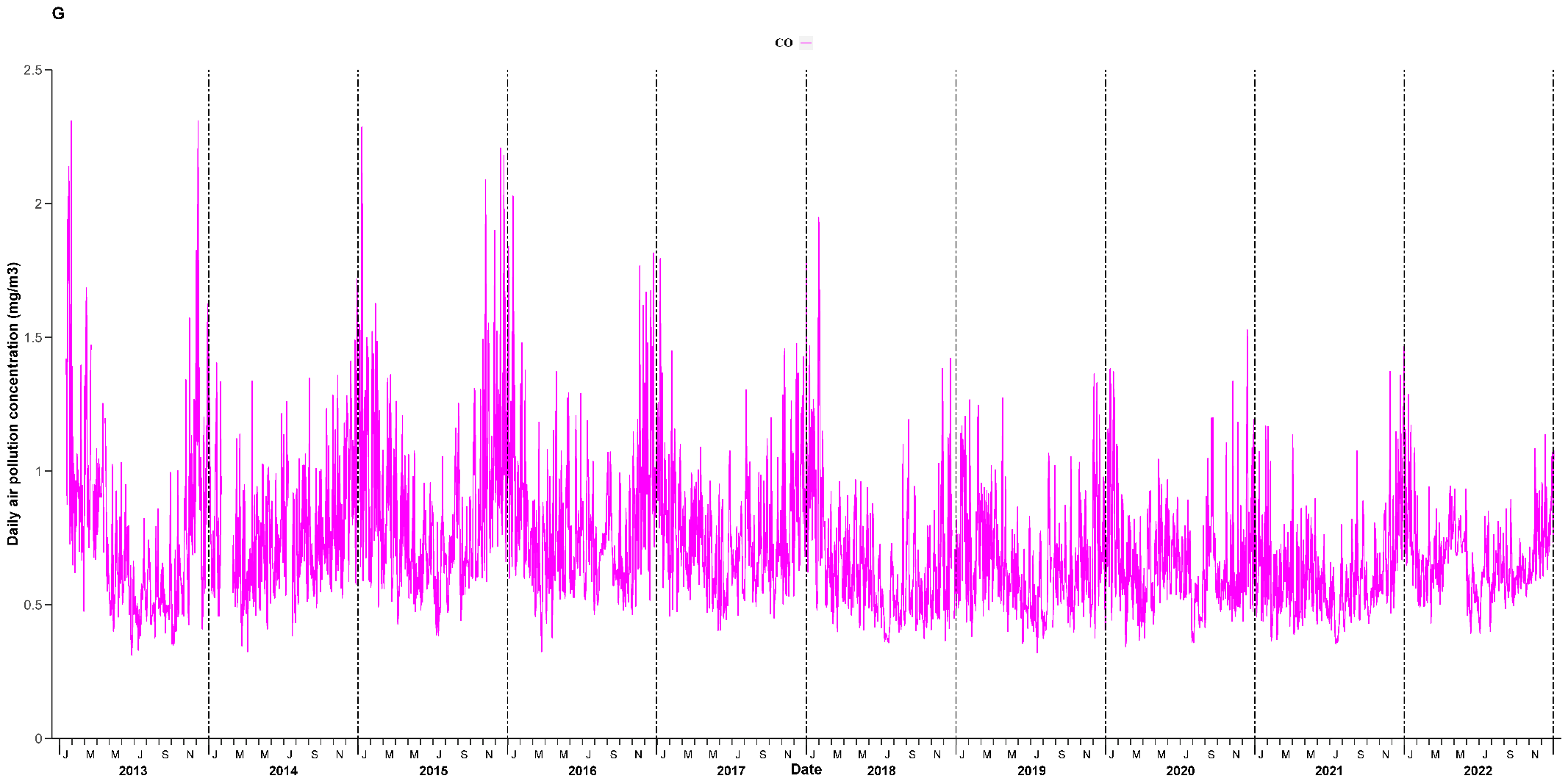


**Figure S3. Time-series plot of air pollution concentrations during January 1, 2013 to December 31, 2022.**

**
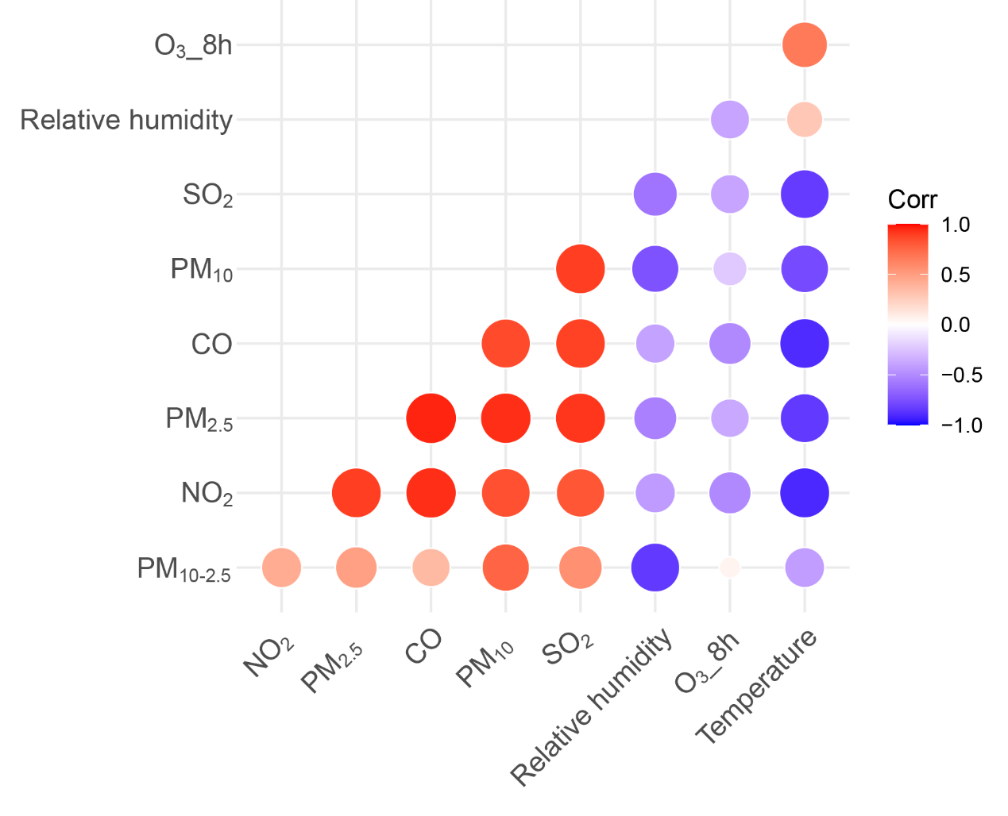
**

**Figure S4. Spearman correlation between air pollutants and weather conditions in Shanghai city during the study period.**


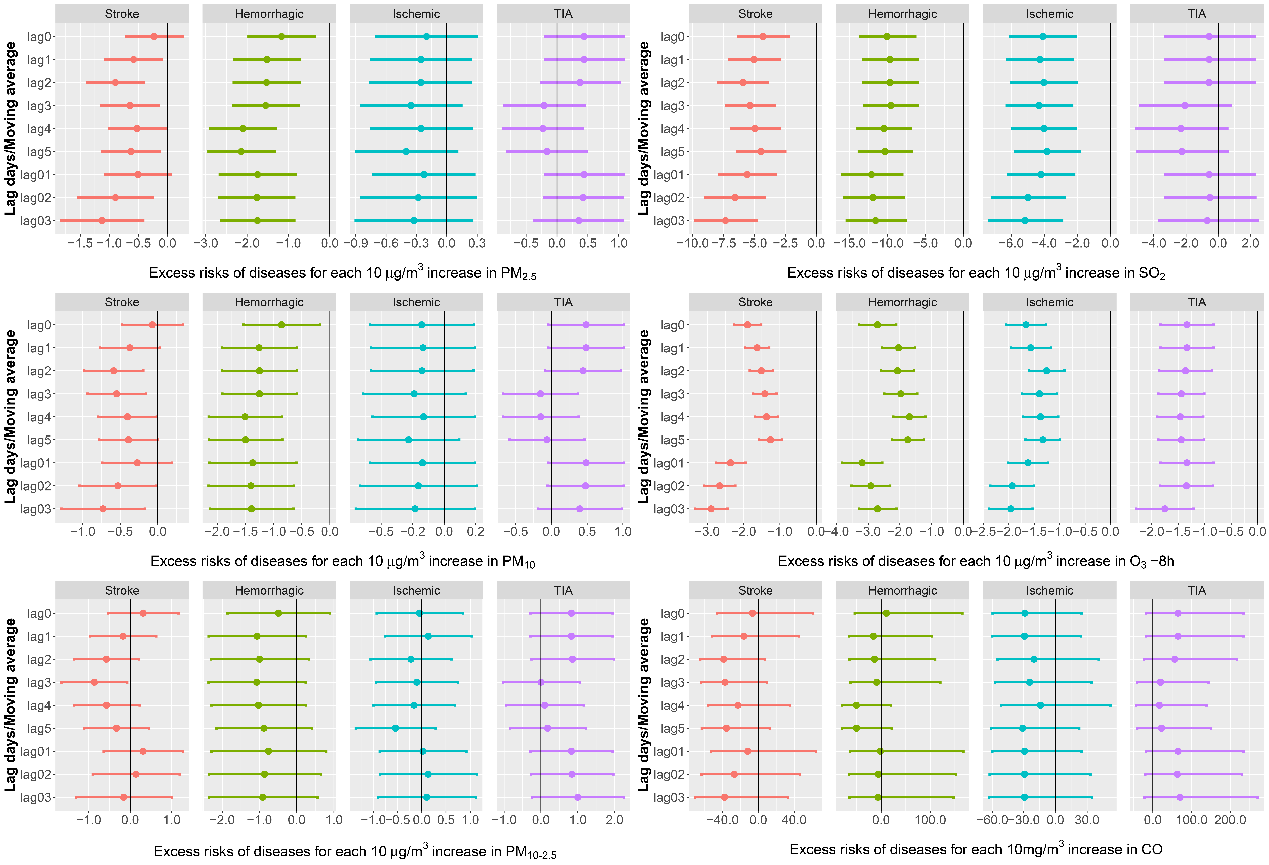


**Figure S5 Forest plots of association between the increase of different pollutants and stroke risk for cases in 2023-2022**


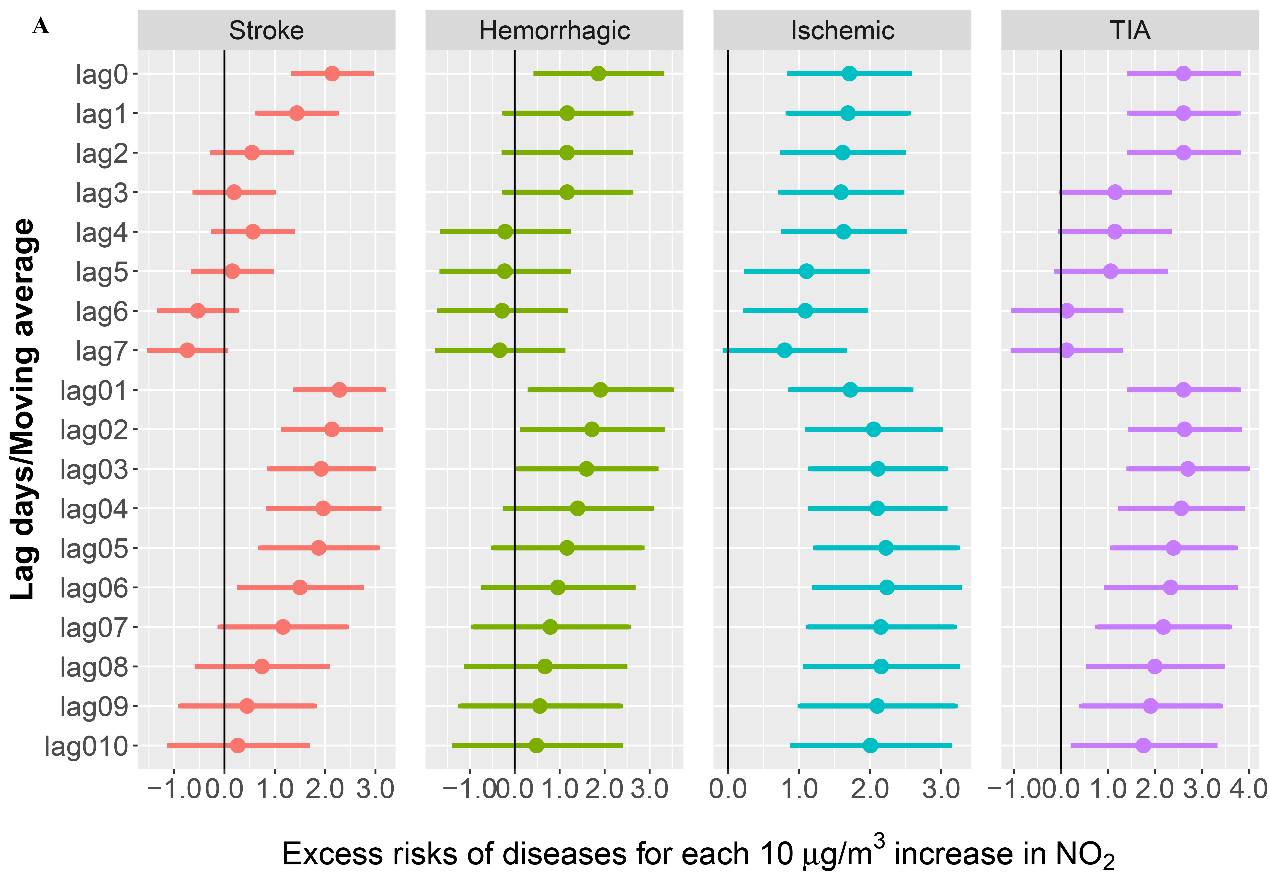


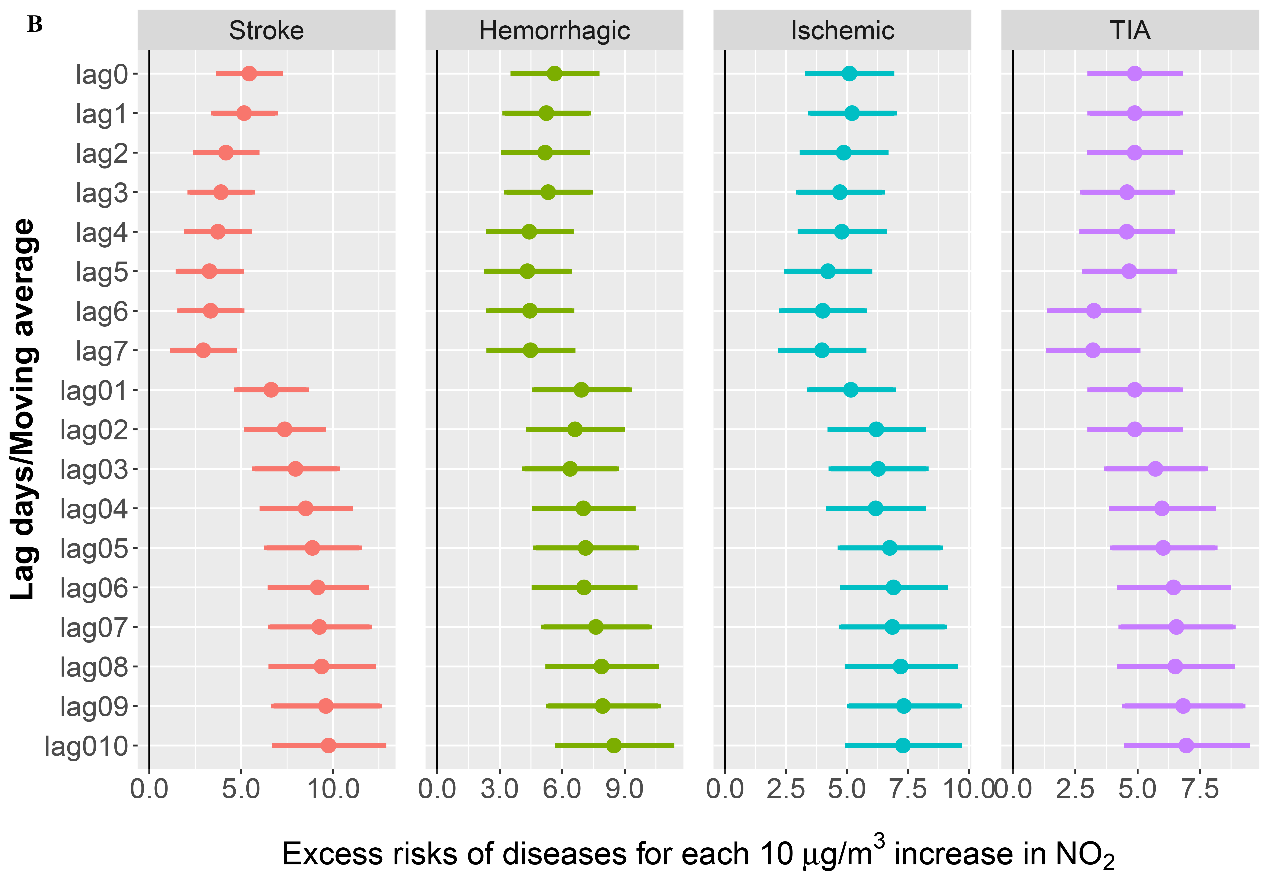


**Figure S6 Forest plots of association between the increase of NO_2_ and stroke risk for cases in 2013–2019 (A) and 2020–2022 (B). We calculated the odds ratios when the daily values of NO_2_ increased by 10 μg/m^3^.**


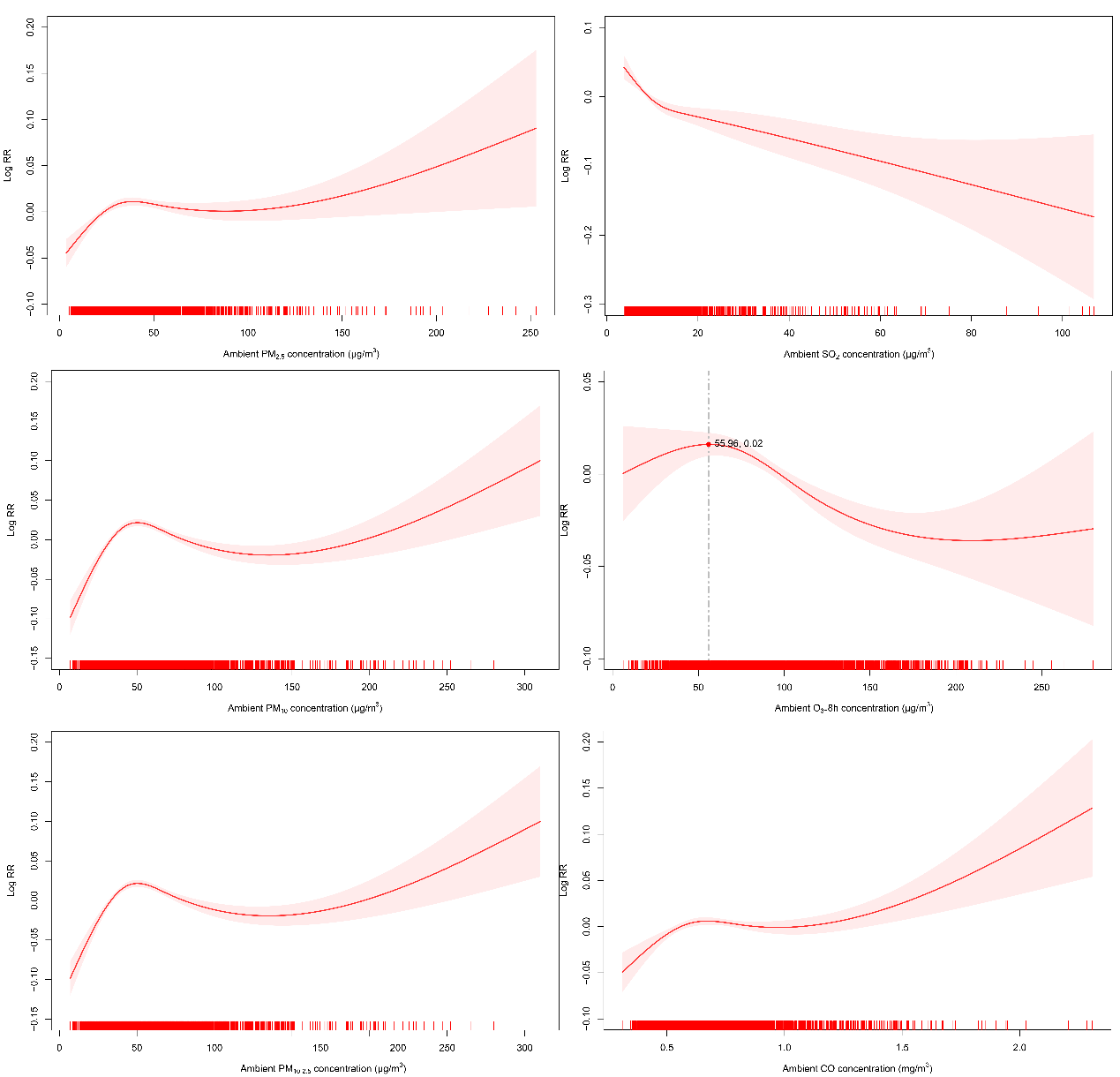


**Figure S7. Exposure-response curve for the association between different pollutants (lag0) and ED visits for stroke in 2013-2022**


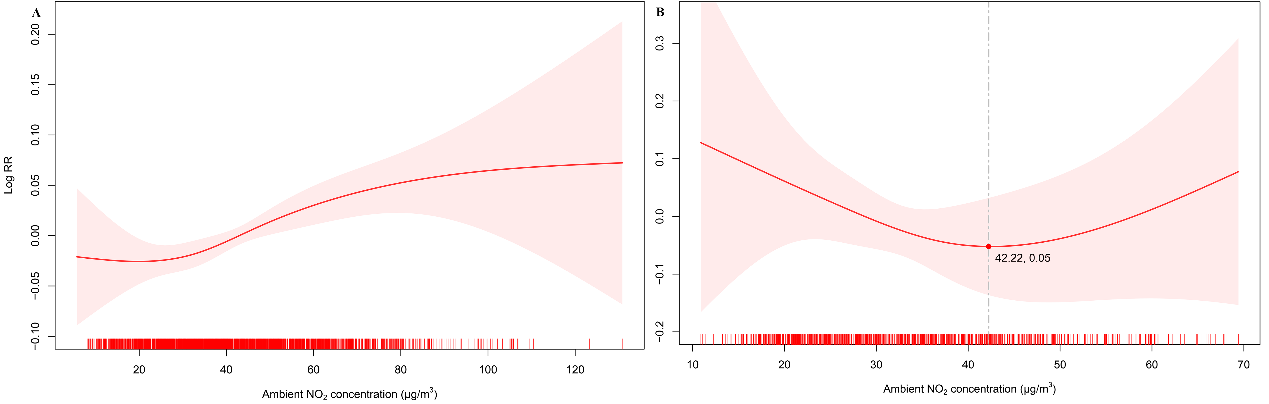


**Figure S8. Exposure-response curve for the association between NO_2_ (lag05) and ED visits for stroke in 2013–2019 (A) and 2020–2022 (B).** The line represents the point estimates and the shading indicates corresponding 95% CIs, which were derived from an over-dispersed generalized additive model, with calendar time, weather conditions, day of the week (DOW) and public holiday (PH) controlled.

Table S1. Spearman correlation between air pollutants and weather conditions in Shanghai city, 2013-2020.

|  | **PM_2.5_** | | **PM_10-2.5_** | **PM_10_** | **SO_2_** | **NO_2_** | **O_3__8h** | **CO** | **Mean temperature** | **Relative humidity** |
| --- | --- | --- | --- | --- | --- | --- | --- | --- | --- | --- |
| **PM_2.5_** | | **1.000** | 0.266 | **0.870** | **0.687** | **0.637** | 0.044 | **0.814** | **-0.281** | -0.193 |
| **PMc** | | 0.266 | **1.000** | **0.644** | 0.342 | 0.251 | 0.129 | 0.178 | -0.078 | **-0.460** |
| **PM_10_** | | **0.870** | **0.644** | **1.000** | **0.655** | **0.611** | 0.106 | **0.697** | **-0.232** | **-0.386** |
| **SO_2_** | | **0.687** | 0.342 | **0.655** | **1.000** | **0.453** | -0.07 | **0.656** | **-0.311** | -0.281 |
| **NO_2_** | | **0.637** | 0.251 | **0.611** | **0.453** | **1.000** | -0.126 | **0.727** | **-0.401** | -0.145 |
| **O_3__8h** | | 0.044 | 0.129 | 0.106 | -0.07 | -0.126 | **1.000** | -0.081 | **0.502** | -0.325 |
| **CO** | | **0.814** | 0.178 | **0.697** | **0.656** | **0.727** | -0.081 | **1.000** | **-0.340** | -0.072 |
| **Mean temperature** | | **-0.281** | -0.078 | **-0.232** | **-0.311** | **-0.401** | **0.502** | **-0.340** | **1.000** | 0.151 |
| **Relative humidity** | | -0.193 | **-0.460** | **-0.386** | -0.281 | -0.145 | -0.325 | -0.072 | 0.151 | **1.000** |

Note: Correlations of statistical significance are in bold.

**Table S2** Excess risk (%) and 95%CI of outpatients for stroke associated with a 10 μg/m^3^ increase in NO_2_ (lag0) under varying degrees of freedom for the smooth functions of calendar time in single-pollutant models.

| **df for time**  **(per year)** | **Stroke** | **Hemorrhagic stroke** | **Ischemic stroke** | **TIA** |
| --- | --- | --- | --- | --- |
| 4 | 3.14 (2.32, 3.96)*** | 2.08 (0.86, 3.31)*** | 3.04 (2.20, 3.88)*** | 2.90 (1.84, 3.98)*** |
| 5 | 3.28 (2.48, 4.08)*** | 2.26 (1.08, 3.44)*** | 3.11 (2.29, 3.94)*** | 3.04 (1.98, 4.11)*** |
| 6 | 3.19 (2.45, 3.94)*** | 2.29 (1.11, 3.47)*** | 2.93 (2.16, 3.70)*** | 3.12 (2.14, 4.12)*** |
| 7 | 3.12 (2.37, 3.89)*** | 2.28 (1.10, 3.47)*** | 2.86 (2.08, 3.64)*** | 3.00 (1.99, 4.01)*** |
| 8 | 3.39 (2.65, 4.13)*** | 2.36 (1.20, 3.53)*** | 3.09 (2.33, 3.86)*** | 3.36 (2.39, 4.33)*** |
| 9 | 3.83 (3.11, 4.55)*** | 2.87 (1.75, 4.00)*** | 3.47 (2.71, 4.23)*** | 3.74 (2.79, 4.69)*** |
| 10 | 3.72 (2.99, 4.46)*** | 2.80 (1.68, 3.93)*** | 3.40 (2.63, 4.17)*** | 3.68 (2.72, 4.64)*** |
| 11 | 3.70 (2.98, 4.41)*** | 2.88 (1.76, 4.02)*** | 3.32 (2.57, 4.08)*** | 3.70 (2.76, 4.66)*** |
| 12 | 3.59 (2.88, 4.30)*** | 2.78 (1.67, 3.91)*** | 3.23 (2.48, 3.98)*** | 3.59 (2.65, 4.54)*** |
| 13 | 3.72 (3.00, 4.44)*** | 2.93 (1.81, 4.05)*** | 3.34 (2.58, 4.10)*** | 3.72 (2.77, 4.68)*** |
| 14 | 3.77 (3.06, 4.49)*** | 3.10 (2.02, 4.19)*** | 3.39 (2.64, 4.15)*** | 3.71 (2.77, 4.67)*** |
| 15 | 3.71 (3.02, 4.40)*** | 2.93 (1.91, 3.95)*** | 3.35 (2.61, 4.09)*** | 3.73 (2.79, 4.67)*** |
| 16 | 3.66 (2.97, 4.35)*** | 2.82 (1.83, 3.83)*** | 3.33 (2.59, 4.07)*** | 3.80 (2.87, 4.74)*** |

Associations of statistically significance are in bold, ***, ** and * indicate p<0.001, p<0.01 and p<0.05, respectively.

**Table S3** Excess risk (%) and 95%CI of outpatients for stroke associated with a 10 μg/m^3^ increase in NO_2_ (lag0) under varying degrees of freedom for the smooth functions of temperature in single-pollutant models.

| **df for temperature** | **Stroke** | **Hemorrhagic stroke** | **Ischemic stroke** | **TIA** |
| --- | --- | --- | --- | --- |
| 4 | 3.42 (2.68, 4.16)*** | 2.39 (1.23, 3.56)*** | 3.10 (2.34, 3.87)*** | 3.40 (2.44, 4.37)*** |
| 5 | 3.42 (2.68, 4.16)*** | 2.39 (1.23, 3.56)*** | 3.10 (2.34, 3.87)*** | 3.40 (2.44, 4.37)*** |
| 6 | 3.39 (2.65, 4.13)*** | 2.36 (1.20, 3.53)*** | 3.09 (2.33, 3.86)*** | 3.36 (2.39, 4.33)*** |
| 7 | 3.40 (2.66, 4.14)*** | 2.36 (1.20, 3.53)*** | 3.10 (2.33, 3.87)*** | 3.36 (2.40, 4.34)*** |
| 8 | 3.40 (2.66, 4.14)*** | 2.36 (1.20, 3.53)*** | 3.10 (2.34, 3.87)*** | 3.37 (2.41, 4.34)*** |

Associations of statistically significance are in bold, *** and ** indicate p<0.001 and p<0.01, respectively.

**Table S4** Excess risk (%) and 95%CI of outpatients for stroke associated with a 10 μg/m^3^ increase in NO_2_ (lag0) under varying degrees of freedom for the smooth functions of relative humidity in single-pollutant models.

| **df for Relative humidity** | **Stroke** | **Hemorrhagic stroke** | **Ischemic stroke** | **TIA** |
| --- | --- | --- | --- | --- |
| 3 | 3.39 (2.65, 4.13)*** | 2.36 (1.20, 3.53)*** | 3.09 (2.33, 3.86)*** | 3.36 (2.39, 4.33)*** |
| 4 | 3.38 (2.65, 4.13)*** | 2.35 (1.19, 3.53)*** | 3.08 (2.31, 3.85)*** | 3.36 (2.40, 4.33)*** |
| 5 | 3.39 (2.65, 4.13)*** | 2.37 (1.21, 3.54)*** | 3.08 (2.31, 3.85)*** | 3.36 (2.40, 4.34)*** |

Associations of statistically significance are in bold, *** and ** indicate p<0.001 and p<0.01, respectively.

**Table S5** Excess risk (%) and 95% CI of outpatients for stroke associated with a 10 μg/m^3^ increase in NO_2_ (lag0) when a longer time period of ambient temperature was controlled

|  | **Stroke** | **Hemorrhagic stroke** | **Ischemic stroke** | **TIA** |
| --- | --- | --- | --- | --- |
| temperature | 3.39 (2.65, 4.13)*** | 2.36 (1.20, 3.53)*** | 3.09 (2.33, 3.86)*** | 3.36 (2.39, 4.33)*** |
| temp03 | 3.90 (3.15, 4.66)*** | 2.67 (1.50, 3.85)*** | 3.19 (2.43, 3.96)*** | 3.52 (2.55, 4.49)*** |
| temp07 | 4.03 (3.27, 4.80)*** | 2.89 (1.70, 4.10)*** | 3.39 (2.61, 4.17)*** | 4.02 (3.04, 5.00)*** |
| temp014 | 4.17 (3.41, 4.94)*** | 2.96 (1.76, 4.18)*** | 3.39 (2.60, 4.19)*** | 4.37 (3.38, 5.36)*** |

temp03 refers to moving average temperature of the current day to three previous days; temp03 refers to moving average temperature of the current day to seven previous days; temp03 refers to moving average temperature of the current day to fourteen previous days. Associations of statistically significance are in bold, ***, ** and * indicate p<0.001, p<0.01 and p<0.05, respectively.

Guo H, Zhang S, Zhang Z, Zhang J, Wang C, Fang X, et al. 2021. Short-term exposure to nitrogen dioxide and outpatient visits for cause-specific conjunctivitis: A time-series study in jinan, china. Atmospheric Environment 247:118211.

Song J, Liu Y, Lu M, An Z, Lu J, Chao L, et al. 2019. Short-term exposure to nitrogen dioxide pollution and the risk of eye and adnexa diseases in xinxiang, china. Atmospheric Environment 218:117001.
